# Translational regulation and protein-coding capacity of the 5′ untranslated region of human *TREM2*

**DOI:** 10.1038/s42003-023-04998-6

**Published:** 2023-06-08

**Authors:** Motoaki Yanaizu, Haruka Adachi, Makoto Araki, Kenji Kontani, Yoshihiro Kino

**Affiliations:** 1 Department of Bioinformatics and Molecular Neuropathology, Meiji Pharmaceutical University, 2-522-1, Noshio, Kiyose-shi, Tokyo, 204-8588 Japan; 2 Department of RNA Pathobiology and Therapeutics, Meiji Pharmaceutical University, 2-522-1, Noshio, Kiyose-shi, Tokyo, 204-8588 Japan; 3 Department of Biochemistry, Meiji Pharmaceutical University, 2-522-1, Noshio, Kiyose-shi, Tokyo, 204-8588 Japan

**Keywords:** Translation, Post-translational modifications, Gene regulation, Neuroimmunology, Evolutionary biology

## Abstract

TREM2 is a transmembrane receptor expressed in microglia and macrophages. Elevated TREM2 levels in these cells are associated with age-related pathological conditions, including Alzheimer’s disease. However, the regulatory mechanism underlying the protein expression of TREM2 remains unclear. In this study, we uncover the role of the 5′ untranslated region (5′-UTR) of human *TREM2* in translation. An upstream start codon (uAUG) in the 5′-UTR of *TREM*2 is specific to some primates, including humans. The expression of the conventional TREM2 protein, starting from the downstream AUG (dTREM2), is repressed by the 5′-UTR in a uAUG-mediated manner. We also detect a TREM2 protein isoform starting from uAUG (uTREM2) that is largely degraded by proteasomes. Finally, the 5′-UTR is essential for the downregulation of dTREM2 expression in response to amino acid starvation. Collectively, our study identifies a species-specific regulatory role of the 5′-UTR in TREM2 translation.

## Abstract (web summary)

An upstream start codon in the 5’UTR of TREM2 specific to some primates and humans initiates translation of an elongated TREM2 isoform and is involved in repressing translation of the conventional TREM2 from the downstream start codon.

## Introduction

Triggering receptor expressed on myeloid cells 2 (TREM2) is a transmembrane protein that acts as a lipid-sensing receptor^1^. TREM2 is predominantly expressed in microglia in the brain. Further, it is involved in microglial phagocytosis, synaptic pruning, and the inflammatory response^2–4^. Rare variants of *TREM2* have been shown to increase the risk of Alzheimer’s disease (AD)^5,6^. Moreover, homozygous mutations in *TREM2* lead to Nasu-Hakola disease, a rare disease characterized by early-onset dementia and bone cysts^7^. Disease-associated variants of *TREM2* impair the substrate-specific functions and survival of microglia^8^. In contrast, the elevated expression of TREM2 or antibody-mediated activation of TREM2 rescues some disease phenotypes in AD model mice^9,10^. TREM2 is essential for the transition from homeostatic microglia to a disease-associated microglial state^11^. Its expression also marks tumor-associated macrophages and lipid-associated macrophages^12,13^. Thus, TREM2 has been implicated in age-related conditions including AD^9–11^, cancer^12^, and obesity^13^. Although the cellular functions of TREM2 have been demonstrated, the regulatory mechanism of *TREM2* remains elusive.

The appropriate expression level of proteins is determined by non-coding elements, such as the 5′ untranslated region (UTR)^14^. Upstream AUG (uAUG) is an evolutionarily conserved feature of the 5′-UTR between humans and rodents^15^. uAUG represses the expression of the protein derived from the downstream main ORF (open reading frame) by disrupting ribosome scanning or inducing nonsense-mediated mRNA decay^16^. Genetic and bioinformatics studies have estimated that ~60% of all human protein-coding genes contain uAUG^17^.

Here, we report the role of uAUG in the 5′-UTR of *TREM2*, located upstream of the downstream AUG (dAUG). Intriguingly, uAUG was determined to be conserved among primates, but not in mice. The 5′-UTR of human *TREM2* was found to repress the expression of conventional TREM2 translated from dAUG (referred to as dTREM2) in a uAUG-dependent manner. In addition, we detected a TREM2 isoform derived from the uAUG. Our study reveals the species-dependent role of the 5′-UTR in *TREM2* translation.

## Results

### Species-specific uAUG in the 5′-UTR of *TREM2*

**Fig. 1:**
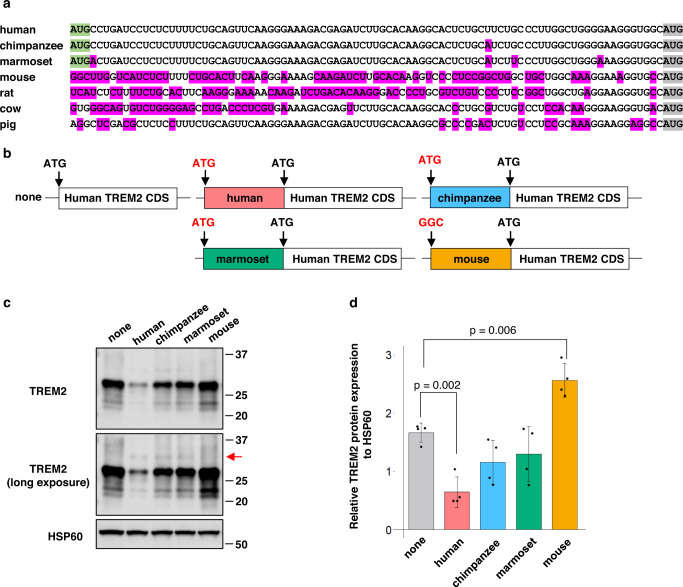
Species-specific upstream AUG (uAUG) of *TREM2* and effect of the 5′-UTR (untranslated region) on protein expression. **a** RNA sequence comparison of 90 bases upstream of downstream AUG (dAUG). The uAUG and dAUG are highlighted in green and gray, respectively. The base substitution is marked in magenta. **b** Schematic diagram of chimeric constructs of the human *TREM2* CDS fused with the 90-base sequence upstream of the dAUG of each species. **c** Results of western blotting using HEK293 cells transfected with each chimeric construct. The red arrow indicates the protein band of uTREM2. **d** Quantitative analysis of TREM2 protein expression in **c**. The results of Tukey’s test are shown. Error bars represent the mean ± SD (*n* = 4).

A sequence comparison revealed the presence of a uAUG, located 90 bases upstream of the dAUG in the 5′-UTR of *TREM2* in most primate species, including humans, but not in other mammals, such as mice (Fig. 1a, Supplementary Fig. 1). Hereafter, we refer to the 90-base region upstream of dAUG as the 5′-UTR for simplicity. Ribosome profiling datasets^18–38^ suggested that ribosomes recognize the initial half of the 5′-UTR of human *TREM2*, but not the corresponding region of mouse *Trem2* (Supplementary Fig. 2a, Supplementary Table 1). We examined the relative usage of uAUG compared to that of dAUG by ribosomes using a ribo-seq dataset^39^ and found that the recognition of uAUG by ribosomes was ~30% of that of dAUG (Supplementary Fig. 2b). Although cell type was different, similar analysis of mouse microglia^40^ suggested a lower fraction (~5%) of Trem2 5′-UTR bound to ribosome (Supplementary Fig. 2b). Thus, we hypothesized that uAUG influences the translation of dTREM2. We prepared expression vectors in which 90-base-long sequences upstream from the dAUG of different species were fused to the coding sequence (CDS) of human *TREM2* (Fig. 1b). The 5′-UTR of human *TREM2* significantly decreased the expression of dTREM2 compared to that with the construct lacking the 5′-UTR of *TREM2* (Fig. 1c, d). In contrast, no such decrease in dTREM2 was observed when the mouse 5′-UTR was fused to this gene (Fig. 1c, d). Meanwhile, the 5′-UTR of chimpanzees and marmosets showed intermediate effects. Notably, a protein band larger than that of dTREM2 was detected when the 5′-UTR of primates was fused to this gene (Fig. 1c, referred to as uTREM2). Translation from uAUG is predicted to produce a TREM2 isoform with an extension of 30 amino acid residues added at the N-terminus of dTREM2. These data suggest that uAUG plays two roles, repression of the expression of dTREM2 and the production of uTREM2.

### The translation of TREM2 is repressed in a uAUG-dependent manner

**Fig. 2:**
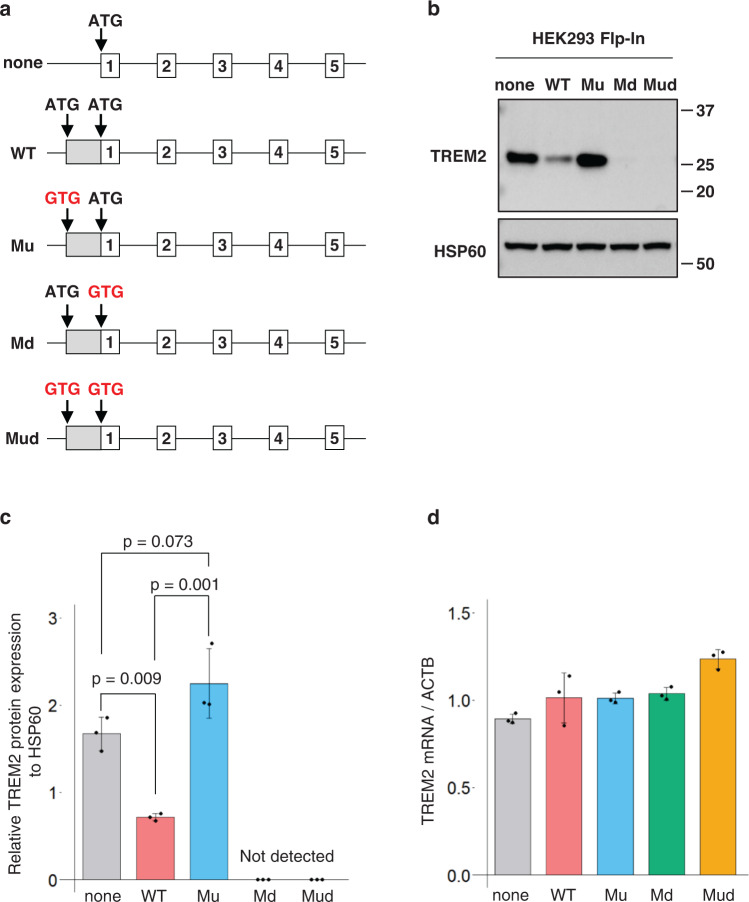
Upstream AUG (uAUG)-dependent repression of TREM2 translation. **a** Schematic diagram of a series of fl-*TREM2* minigene constructs with or without the 5′-UTR (untranslated region). Mutated initiation codons are indicated in red. HEK293 cells stably expressing these minigenes were established using the Flp-In system. **b** Total cell lysates from each stable cell line were resolved by SDS-PAGE and probed with an anti-TREM2 antibody. **c** Quantification of western blotting results. Data represent the mean ± SD (*n* = 3, Tukey’s test). **d** *TREM2* mRNA levels in each cell line normalized to *ACTB*. Error bars indicate the mean ± SD (*n* = 3, Tukey’s test). No significant differences were detected between the 5′-UTR-none and 5′-UTR-WT cells.

To further elucidate the role of uAUG, we established isogenic cell lines using the HEK293-based Flp-In system, to stably express a full-length human *TREM2* minigene containing the 5′-UTR in which the uAUG and/or dAUG were mutated (Fig. 2a). Western blot analysis of total cell lysates showed a decrease in dTREM2 derived from the 5′-UTR-WT cell line compared with that from 5′-UTR-none (Fig. 2b, c). In contrast, the expression level of dTREM2 derived from 5′-UTR-Mu was comparable to that derived from 5′-UTR-none (Fig. 2b, c). No TREM2 protein bands were observed in the 5′-UTR-Md and 5′-UTR-Mud cell lines (Fig. 2b). Thus, the expression level of the dTREM2 protein was regulated in a uAUG-dependent manner, whereas *TREM2* mRNA derived from 5′-UTR-WT, 5′-UTR-Md, and 5′-UTR-Mud did not show any reduction compared to the level from 5′-UTR-none (Fig. 2d). Therefore, uAUG is essential for the repression of dTREM2 by the 5′-UTR at the translational level.

### Detection of uTREM2 as an endogenous protein

**Fig. 3:**
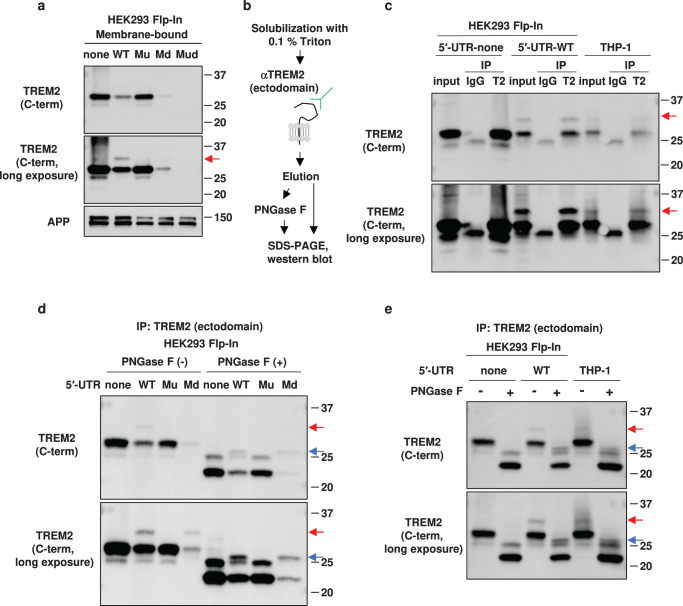
Detection of uTREM2 (TREM2 protein isoform starting from upstream AUG) by cell fractionation and immunoprecipitation. **a** Membrane-bound protein fractions of stable cell lines were subjected to western blot analysis. The red arrow indicates the protein band of uTREM2. APP was used as a loading control. **b** Scheme of immunoprecipitation and PNGase F treatment. **c** Immunoprecipitation using anti-TREM2 antibodies in the indicated cells. The red arrows indicate uTREM2. **d** 5′-UTR (untranslated region)-none, 5′-UTR-WT, 5′-UTR-Mu, and 5′-UTR-Md cells were subjected to immunoprecipitation followed by deglycosylation with PNGase F. **e** TREM2 expressed in THP-1 cells was immunoprecipitated followed by treatment with PNGase F. Red and blue arrows indicate the protein band of uTREM2 and deglycosylated uTREM2, respectively (**d**, **e**).

As shown in Fig. 2b, uTREM2 could barely be detected in the total cell lysates of 5′-UTR-WT and 5′-UTR-Md cells. We then investigated the optimal conditions for detecting uTREM2 expression. The use of 0.1% Triton-X-100 for cell lysis and an antibody against the C-terminus of TREM2 for immunoblotting facilitated the detection of the protein band corresponding to uTREM2 in the 5′-UTR-WT cells (red arrow, Supplementary Fig. 3). Furthermore, uTREM2 was clearly detected when the membrane-bound protein fraction was used (Fig. 3a). THP-1 cells produced a protein band of endogenous uTREM2 only when these cells were treated with phorbol-myristate-acetate (PMA) (Supplementary Fig. 3). The uTREM2 band was detected in the membrane-bound protein fraction of 5′-UTR-WT, but not in the other cells (Fig. 3a).

To examine the integrity of uTREM2, 5′-UTR-none, 5′-UTR-WT, and THP-1 cells were immunoprecipitated with an antibody against the ectodomain of dTREM2 and probed with another antibody against its C-terminus (Fig. 3b). Both 5′-UTR-WT and THP-1 cells resulted in a protein band of uTREM2 in the immunoprecipitated fraction (red arrows, Fig. 3c). This also indicated that uTREM2 shares ectodomain and C-terminal sequences with dTREM2. To confirm whether uTREM2 contains specific N-terminal peptides, the protein band of uTREM2 from the 5′-UTR cells was subjected to liquid chromatography-tandem mass spectrometry (LC-MS/MS) analysis following immunoprecipitation. We consequently detected a peptide, MPDPLFSAVQGK, which corresponded to the unique N-terminus of uTREM2 (Supplementary Fig. 4), supporting our hypothesis that translation from uAUG leads to the production of uTREM2 protein.

TREM2 is subjected to glycosylation as a post-translational modification^41^. To verify that uTREM2 was glycosylated, the immunoprecipitated products derived from our stable cell lines were deglycosylated with PNGase F (Fig. 3b). In the absence of PNGase F, immunoprecipitates from both 5′-UTR-WT and 5′-UTR-Md cells showed a protein band of uTREM2 (red arrows, Fig. 3d). Thus, uTREM2 was expressed in the 5′-UTR-Md cells, and the lack of its detection in previous experiments could be due to the low level of uTREM2. After PNGase F treatment, both 5′-UTR-none and 5′-UTR-Mu showed similar patterns (Fig. 3d). Notably, a unique protein band was detected in the 5′-UTR-WT and 5′-UTR-Md cells, which was absent in the 5′-UTR-none and 5′-UTR-Mu cells and should be derived from the uAUG (Fig. 3d, indicated by blue arrows), implying that uTREM2 is subjected to glycosylation (Fig. 3d). Furthermore, THP-1 cells showed a protein band pattern similar to that of 5′-UTR-WT (Fig. 3e). These results suggested that uTREM2 is glycosylated.

### Translation from the uAUG is required for the expression of uTREM2

**Fig. 4:**
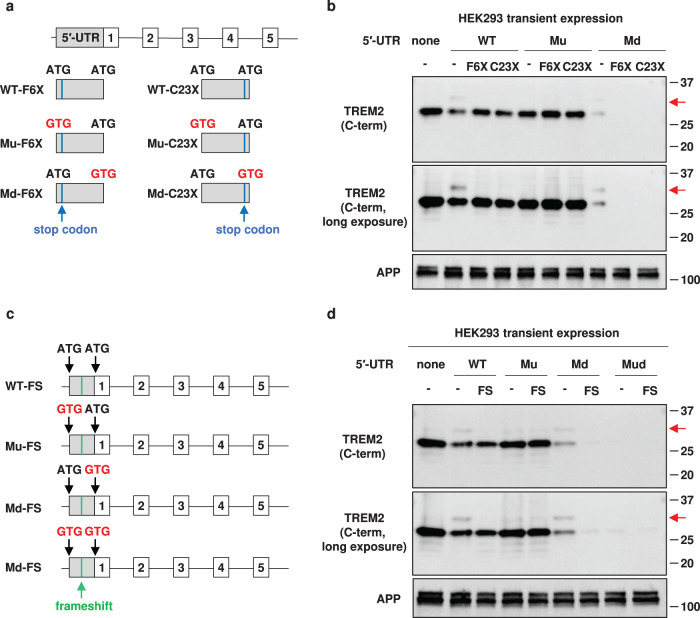
Translation depending on upstream AUG (uAUG) is required for the expression of uTREM2 (TREM2 protein isoform starting from uAUG). **a** Blue lines represent stop codons introduced between uAUG and downstream AUG (dAUG). F6X and C23X represent stop codons introduced into the 5′-UTR (untranslated region) of *TREM2*. **b** Membrane-bound fractions of HEK293 cells transfected with these minigenes were analyzed by western blotting. APP was used as a loading control. The red arrows indicate uTREM2. **c** Schematic diagram of frameshift mutants. A frameshift in minigenes was induced by a single-nucleotide insertion in the middle of the 5′-UTR. **d** Membrane fractions of HEK293 cells transfected with frameshift minigenes were subjected to western blot analysis using an anti-TREM2 antibody.

To verify that uTREM2 is translated from uAUG, we prepared a series of fl-*TREM2* mutant minigenes with a stop codon in the 5′-UTR (Fig. 4a). As uAUG is the first codon of uTREM2, the substitution comprising the 6th and 23rd codons from uAUG was denoted as F6X and C23X, respectively. The protein band of uTREM2 was abolished in both the 5′-UTR-WT-F6X and 5′-UTR-WT-C23X minigenes (red arrows, Fig. 4b). Simultaneously, the expression level of dTREM2 was partially restored with these constructs, suggesting that continuous translation from uAUG to dAUG is required for the repressive regulation of TREM2 translation. The level of dTREM2 protein derived from the 5′-UTR-Mu-F6X and 5′-UTR-WT-C23X minigenes was comparable to that from the 5′-UTR-Mu minigene (Fig. 4b). Moreover, protein bands of both uTREM2 and dTREM2 were detected with the 5′-UTR-Md minigene; however, no protein band was detected with the 5′-UTR-Md-F6X and 5′-UTR-Md-C23X minigenes (Fig. 4b). This strongly indicates that both bands were derived from the uAUG-initiated translation of the 5′-UTR-Md minigene.

We also prepared a full-length minigene with a 5′-UTR in which a frameshift was introduced by a single-nucleotide insertion (Fig. 4c, referred to as FS). The expression of uTREM2 was also disrupted with the 5′-UTR-WT-FS minigene (Fig. 4d). Remarkably, the expression level of dTREM2 derived from 5′-UTR-WT-FS was comparable to that from 5′-UTR-WT (Fig. 4d), indicating that the frameshift mutation did not increase dTREM2 expression. This was in contrast to the above observation (Fig. 4b) in which dTREM2 of 5′-UTR-WT was increased by introducing point mutations (F6X or C23X). These observations are in line with the fact that continuous translation from uAUG that passes through dAUG could contribute to the repression of dTREM2. The protein bands of both uTREM2 and dTREM2 were abrogated with the 5′-UTR-Md-FS minigene (Fig. 4d). Together with the observation shown in Fig. 4b, these results raise the possibility that uTREM2 is partially subjected to digestion at the signal peptide and produces the dTREM2 protein. There was no difference in the intracellular localization of TREM2 among these mutants (Supplementary Fig. 5).

### The 5′-UTR of *TREM2* mediates the repression of TREM2 protein expression in response to stress

**Fig. 5:**
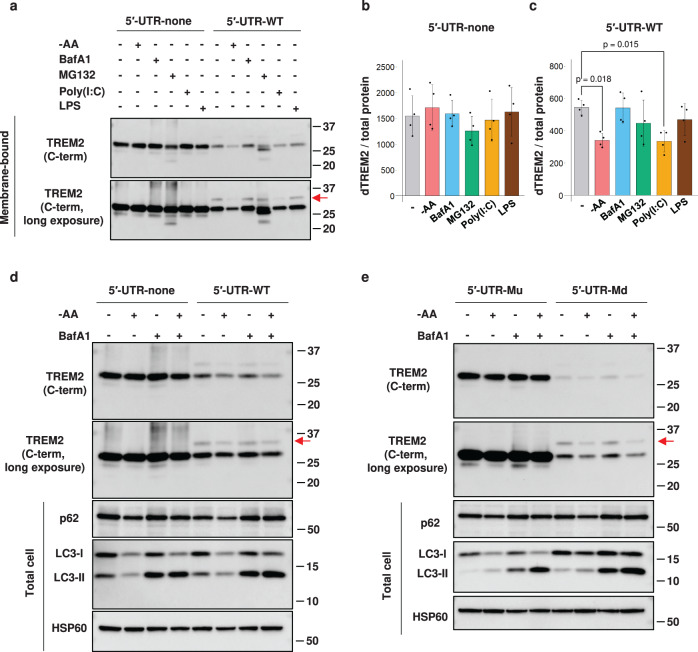
The 5′-UTR (untranslated region) of TREM2 mediates translational responses to stresses. **a** The 5′-UTR-none and WT cell lines were exposed to cellular stress. The fractions of membrane-bound proteins were subjected to western blotting using the indicated antibodies. The red arrow indicates uTREM2 (TREM2 protein isoform starting from upstream AUG). **b**, **c** Quantification of downstream TREM2 (dTREM2) from 5′-UTR-none cells (**b**) and 5′-UTR-WT cells (**c**) based on the western blot results. Differences from the untreated control were analyzed using Dunnett’s test. Error bars represent the mean ± SD (*n* = 4). **d**, **e** Stable cell lines were cultured in a starvation medium with bafilomycin A1. Total cell lysates were used to confirm the effects of the treatment.

Next, we investigated the significance of the 5′-UTR of *TREM2* in cell physiology by comparing the response of TREM2 expression to cellular stress (Fig. 5a, Supplementary Fig. 6a). The cellular stress conditions tested in this study were as follows: (i) amino acid starvation; (ii) bafilomycin A1 (BafA1), a vacuolar H + -ATPase inhibitor; (iii) a proteasome inhibitor, MG132; (iv) polyinosinic-polycytidylic acid (polyI:C), a synthetic analog of double-stranded RNA (dsRNA); and (v) lipopolysaccharide (LPS), a mediator of inflammation. The expression level of dTREM2 derived from the 5′-UTR-none cells was not altered under the conditions examined (Fig. 5b). Intriguingly, a band slightly smaller than dTREM2 appeared specifically in the 5′-UTR-WT cells with MG132 treatment (Fig. 5a), suggesting the presence of a 5′-UTR-derived TREM2 protein that was degraded thoroughly by the proteasome. Moreover, the expression level of dTREM2 in 5′-UTR-WT cells was significantly reduced by amino acid starvation and polyI:C treatment (Fig. 5a, c). BafA1 and LPS treatment did not alter the expression levels of TREM2 (Fig. 5a, c). However, simultaneous treatment with amino acid starvation and BafA1 reduced the expression level of dTREM2 in the 5′-UTR-WT (Fig. 5d), demonstrating that the reduction in dTREM2 mediated by amino acid starvation was not due to enhanced degradation via autophagy. The expression level of dTREM2 in 5′-UTR-Mu cells was not altered by amino acid starvation, whereas that in 5′-UTR-Md cells was decreased (Fig. 5e), indicating that uAUG is essential for the downregulation of both uTREM2 and dTREM2 mediated by amino acid starvation. Endogenous dTREM2 protein in THP-1 cells exhibited a similar downregulation upon amino acid starvation (Supplementary Fig. 6b). Finally, we analyzed intracellular localization of TREM2 by immunofluorescence. While the TREM2 signal of the 5′-UTR-none and 5′-UTR-Mu cell lines was obtained, that of the 5′-UTR-WT and 5′-UTR-Md cells was nearly undetectable (Supplementary Fig. 7). We thus transiently expressed *TREM2* minigenes and observed TREM2 localization. Although TREM2 expression was still low in cells transfected with 5′-UTR-Md, the pattern of localization was similar among all four constructs, with little changes induced by different treatments (Supplementary Fig. 8).

### uTREM2 is degraded by proteasomes

**Fig. 6:**
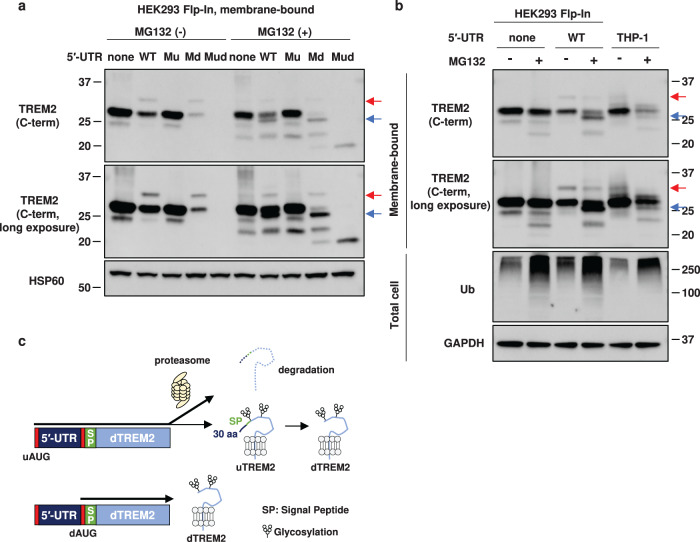
The upstream AUG (uAUG)-derived TREM2 protein is degraded by the proteasome. **a** Total cell lysates of stable cell lines were treated with MG132 and analyzed by western blotting. Ubiquitin (Ub) was used as a positive control for MG132 treatment. Red and blue arrows indicate the protein band of intact uTREM2 and a proteasome-sensitive form of uTREM2, respectively. **b** Western blot analysis of the membrane-bound protein fraction of MG132-treated cells. **c** Working hypothesis of uTREM2 (TREM2 protein isoform starting from uAUG) processing. A large fraction of uTREM2 derived from uAUG is degraded by the proteasome. The remaining uTREM2 fraction is glycosylated and cleaved to produce dTREM2 (TREM2 protein isoform starting from downstream AUG).

As shown in Fig. 5a, MG132 treatment resulted in a band slightly smaller than dTREM2 in 5′-UTR-WT, but not 5′-UTR-none, cells. This band was also detected in 5′-UTR-Md cells upon MG132 treatment (Fig. 6a), strongly suggesting that it was derived from uAUG. A similar band was detected in the membrane fraction of MG132-treated THP-1 cells, albeit at a lower level (Fig. 6b). Thus, it appears that the majority of uAUG-derived products are degraded by proteasomes.

## Discussion

In this study, we revealed a repressive regulatory effect on human *TREM2* translation mediated by its 5′-UTR. We also showed the involvement of the 5′-UTR in the response to amino acid starvation. As these features are dependent on uAUG, which is shared by some primates, our results highlight the species-specific features of TREM2. We found that the uAUG-mediated regulation of *TREM2* translation is conserved in some primates, but not in mice (Fig. 1). Although human and mouse TREM2 proteins are expected to have conserved functions, it is likely that these two proteins are subjected to divergent regulatory mechanisms. For example, we recently reported that TREM2 undergoes the alternative splicing of exon 3^42^. A single-nucleus transcriptome revealed both conserved and non-conserved responses between human and mouse microglia in the context of AD^43^. Thus, our findings could contribute to the understanding of the species-specific aspects of microglia. Recently, upregulation of Trem2 translation in a mouse model of AD has been reported^44^. Because uAUG of *TREM2* that represses dTREM2 translation is absent in mouse *Trem2*, the activation of Trem2 translation in the AD model mice may occur through a mechanism distinct from what we have described. Further investigation is needed to determine the significance of these findings in AD patients. Our mutation analyses also suggested that the continuous translation from uAUG through dAUG is involved in the repression of dTREM2 expression (Fig. 4), which can be interpreted as competition between uAUG and dAUG for translation initiation. We also found that dTREM2 levels are reduced by amino acid starvation and poly(I:C) treatment, depending on the uAUG (Fig. 5), suggesting the involvement of additional determinants^45^. The use of dAUG might be limited by several factors in the presence of uAUG, including disturbances in ribosomal scanning mediated by translation initiation at the uAUG, the secondary structure of the 5′-UTR, and trans-acting factors that bind to the 5′-UTR^46^. Elucidating the precise mechanism of 5′-UTR-mediated repression would provide a strategy to modulate the expression of TREM2.

In addition to translational repression, the *TREM2* 5′-UTR is also involved in the expression of uTREM2. The 30-residue extension at the N-terminus of uTREM2 increases the length of the signal peptide. Unexpectedly, we observed a protein band of dTREM2 together with that of uTREM2 in 5′-UTR-Md cells (Fig. 4b). Both bands disappeared when a stop codon was introduced downstream of the uAUG (Fig. 4b). We propose that the N-terminal region of uTREM2 is cleaved at the same site as the signal peptide of dTREM2, resulting in the production of dTREM2. The N-terminal extension of uTREM2 elongates the signal peptide from 18 to 48 amino acids. Although the average length of signal peptides in eukaryotes is 22 residues^47^, several genes have a long signal peptide of 40 or more residues^48^. Remarkably, we detected a protein band reminiscent of deglycosylated uTREM2, specifically in the 5′-UTR-WT and 5-UTR-Md cells upon proteasome inhibition (Fig. 6a, Supplementary Fig. 9). We speculate that a major fraction of uTREM2 is rapidly degraded by proteasomes and that the remaining fraction is glycosylated and cleaved to produce dTREM2 (Fig. 6c). There is another example of a glycosylated membrane protein (US11) that retains its signal peptide owing to its delayed cleavage^49^. The long signal peptide in uTREM2 might have resulted in the preferential proteasomal degradation of uTREM2 and delayed cleavage of the signal peptide. Although uTREM2 could be regarded as an intermediate of an alternative pathway of dTREM2 production, or as a by-product of the uAUG-mediated repression of dTREM2, further studies are needed to determine its functionality.

In conclusion, this study revealed the role of the 5′-UTR in the translation of human TREM2. As the uAUG-mediated regulatory mechanism of *TREM2* is not conserved in mice, our study emphasizes the importance of studies using human-derived cells to obtain new insights into TREM2-related disorders. Regulation of TREM2 at the translational level is also important, which might be overlooked by transcriptome analyses.

## Materials and methods

### Plasmids

Primers used in this study are listed in Supplementary Table 2. All engineered plasmids were subjected to DNA sequencing.

### Human TREM2 CDS fused with the 5′-UTR of different species

The 5′-UTR from chimpanzees, marmosets, and mice was amplified from the genomic DNA (obtained from HSP-239, HSCj-110^50^, and 91062702, respectively) using specific primers. Fragments of the 5′-UTR and human *TREM2* CDS^42^ were combined by PCR, digested with BamHI and XbaI, and cloned into the BamHI-XbaI site of pcDNA3.1 Hygro (+) (Invitrogen).

### Full-length *TREM2* minigenes containing 5′-UTR

A series of 5′-UTR sequences of human *TREM2* with a mutated uAUG and/or dAUG was fused into the fl-*TREM2* minigene, as described previously^51^. To obtain the 5′-UTR with or without a mutation in uAUG and/or dAUG, the 5′-UTR-fused *TREM2* CDS and fl-*TREM2* minigene were used as PCR templates to amplify the region from the 5′-UTR to the middle of intron 1. The replacement of ATG with GTG was then performed using PCR primers. These two fragments were combined by PCR-mediated amplification, digested with NheI and HindIII, and cloned into the NheI-HindIII site of the fl-*TREM2* minigene.

### *TREM2* minigenes containing a termination codon or a frameshifting insertion in the 5′-UTR

F6X and C23X substitutions (residue numbers were counted from the uAUG) and a frameshifting insertion of a single-nucleotide were introduced by PCR using primers harboring the corresponding mutation. Each fragment was digested with NheI and HindIII and then cloned into the NheI-HindIII site of the fl-*TREM2* minigene containing the 5′-UTR of *TREM2*. PCR for plasmid construction was performed using KOD plus neo (TOYOBO).

### Cell culture and cell line establishment

HEK293 and HeLa cells were maintained in Dulbecco’s modified Eagle’s medium (DMEM, Thermo Fisher Scientific), supplemented with 10% fetal bovine serum (Sigma) and penicillin/streptomycin (Wako) at 37 °C with 5% CO_2_. The HSCj-110 cell line, derived from *Callithrix jacchus* (JCRB1655), the human-derived THP-1 line, and the HSP-239 cell line derived from *Pan troglodytes* (JCRB1165), were obtained from JCRB^50^ and maintained in RPMI 1640 medium (GlutaMAX supplement, Thermo Fisher Scientific) supplemented with 10% fetal bovine serum and penicillin/streptomycin at 37 °C with 5% CO_2_. A stable cell line expressing the fl-*TREM2* minigene without the 5′-UTR (5′-UTR-none) was previously established^51^. Cell lines stably expressing fl-*TREM2* minigenes (5′-UTR-WT, Mu, Md, and Mud) were established using the Flp-In system as described in our previous study^51^. The integration of fl-*TREM2* minigenes was confirmed by PCR, using fragment-specific primers.

### Cell treatment

Cells were treated under the following conditions: DMEM (high glucose) with sodium pyruvate, without amino acids (Wako) for 4 h, 100 nM bafilomycin (Merck) for 4 h, 10 μM MG132 (Sigma) for 12 h, 2 μg mL^−1^ Poly(I:C) (Tocris Bioscience) for 24 h using Lipofectamine RNAiMAX (Thermo Fisher Scientific), and 1 μg mL^−1^ LPS (Sigma) for 6 h. HEK293 cells were seeded on 12-well plates before the day of plasmid DNA transfection. Typically, 0.5 μg of plasmid was transfected into HEK293 cells using Lipofectamine 2000 (Thermo Fisher Scientific) and harvested 48 h after plasmid transfection. Lipofectamine 3000 (Thermo Fisher Scientific) was used for the transfection of the plasmids into HeLa cells.

### Quantitative PCR

Total RNA purification and quantitative analysis were performed as previously described^52^. *ACTB* was used as the reference gene.

### Cell fractionation and western blotting

Cell fractionation and western blotting were performed as in previous studies^42^. Blotting images were captured using a Luminograph III (ATTO). Signal intensities were analyzed using Fiji software (NIH). As shown in Fig. 5, the membrane was stained with Ponceau S solution to measure the total protein prior to blocking and antibody treatment. The antibodies used for western blotting are listed in Supplementary Table 3. The full blot images are shown in Supplementary Fig. 10.

### LC-MS/MS analysis

Protein identification was performed as previously described^53^ with minor changes. Briefly, immunoprecipitation samples were separated by SDS-PAGE and the gels were stained with the Silver Stain MS Kit (Wako). Protein bands were excised from the gel and de-stained with a de-staining solution. Subsequently, the gel was reduced with 25 mM dithiothreitol and alkylated with 1% (w/v) iodoacetamide in 25 mM ammonium bicarbonate. Each gel was trypsinized overnight at 37 °C in Digestion buffer [50 mM ammonium bicarbonate, 0.01% (w/v) ProteaseMax Surfactant (Promega), 1.33 μg ml^−1^ Trypsin (Promega), and 1.33 μg ml^−1^ Lysyl Endopeptidase (Wako)], and the supernatant was retained. Peptides were extracted from the excised gel portions with 2.5% trifluoroacetic acid after shaking for 15 min. After digestion, the resultant peptides were desalted, and concentrated using StageTips (Thermo Fisher). The gradient program using mobile phases A and B (acetonitrile containing 0.1% formic acid) had the following sequence: 0% B (0 min)–30% B (50 min)–100% B (51 min)–100% B (60 min). LC-MS/MS data were processed with Proteome Discoverer version 2.4 (Thermo Fisher Scientific). We added the amino acid sequence of uTREM2 to the Uniprot database (version 04/2017, with 20,198 sequences).

### Immunofluorescence

Immunofluorescence was performed according to methods described in our previous study^42^. Briefly, HeLa cells and HEK Flp-In stable cell lines were cultured in eight-well chamber slide (WATSON). The cells were fixed with 4% paraformaldehyde (Wako) for 10 min and treated with 0.1% Triton for 5 min. After 3 h of incubation with anti-TREM2 C-term antibody, the cells were treated with Alexa Fluor 488 goat anti-rabbit Ig (H + L) or Alexa Fluor 568 donkey anti-rabbit IgG (H + L) (Thermo Fisher Scientific) for 1 h. Cells were mounted with Vectashield mounting medium with DAPI (VECTOR LABORATORIES). Cell images were obtained using confocal microscopy (LSM710; Carl Zeiss).

### Immunoprecipitation

For immunoprecipitation, a goat anti-human TREM2 antibody (R&D, AF1828) was mixed with protein G-conjugated magnetic beads (Thermo Fisher Scientific). Cells were lysed in 0.1% Triton-X in PBS with 1× protease inhibitor (Roche), followed by rotation at 4 °C for 30 min. After centrifugation (16,000 × *g* at 4 °C for 10 min), the supernatant was pre-cleared with protein G-conjugated magnetic beads at 4 °C for 2 h and immunoprecipitated with magnetic beads conjugated with an anti-TREM2 antibody or isotype control by rotating it overnight at 4 °C. Beads were washed three times with 0.1% Triton-X in PBS. Immunoprecipitates were eluted from the beads in 0.1 M Gly-HCl buffer (pH 2.5, containing 0.15 mM NaCl). A rabbit anti-TREM2 antibody (CST, D8I4C) was used as the primary antibody for western blotting. TrueBlot anti-IgG HRP (ROCKLAND) was used as the secondary antibody.

### Deglycosylation

The immunoprecipitated products with anti-TREM2 antibodies were subjected to deglycosylation using PNGase F (NEB) according to the manufacturer’s instructions.

### Statistics and reproducibility

EXCEL Toukei software (ESUMI Co., Ltd.) was used for statistical analysis. All graphs were produced using R software (version 3.6.1, https://www.r-project.org/). The sample sizes are defined in the figure legends. Measurements were taken from distinct biological replicates representing independent transfections or cell treatments. Error bars represent the standard deviation (SD). All statistical tests were two-sided and are described in the figure legends.

### Reporting summary

Further information on research design is available in the Nature Portfolio Reporting Summary linked to this article.

## Supplementary information

Supplementary Information

Description of Additional Supplementary Files

Supplementary Data 1

Reporting Summary

## Data Availability

Source data are available in Supplementary Data 1. Full blots are shown in the Supplementary Information. All other data are available from the corresponding authors upon request.

## References

[1] Wang Y (2015). TREM2 lipid sensing sustains the microglial response in an Alzheimer’s disease model. Cell.

[2] Zhao Y (2018). TREM2 is a receptor for beta-amyloid that mediates microglial function. Neuron.

[3] Filipello F (2018). The microglial innate immune receptor TREM2 is required for synapse elimination and normal brain connectivity. Immunity.

[4] Liu W (2020). Trem2 promotes anti-inflammatory responses in microglia and is suppressed under pro-inflammatory conditions. Hum. Mol. Genet..

[5] Guerreiro R (2013). TREM2 variants in Alzheimer’s disease. N. Engl. J. Med..

[6] Jonsson T (2013). Variant of TREM2 associated with the risk of Alzheimer’s disease. N. Engl. J. Med..

[7] Paloneva J (2002). Mutations in two genes encoding different subunits of a receptor signaling complex result in an identical disease phenotype. Am. J. Hum. Genet..

[8] Garcia-Reitboeck P (2018). Human induced pluripotent stem cell-derived microglia-like cells harboring TREM2 missense mutations show specific deficits in phagocytosis. Cell Rep..

[9] Lee CYD (2018). Elevated TREM2 gene dosage reprograms microglia responsivity and ameliorates pathological phenotypes in Alzheimer’s disease models. Neuron.

[10] Wang, S. et al. Anti-human TREM2 induces microglia proliferation and reduces pathology in an Alzheimer’s disease model. *J. Exp. Med.* **217**, e20200785 (2020). 10.1084/jem.20200785 PMC7478730 32579671

[11] Keren-Shaul H (2017). A unique microglia type associated with restricting development of Alzheimer’s disease. Cell.

[12] Katzenelenbogen Y (2020). Coupled scRNA-Seq and intracellular protein activity reveal an immunosuppressive role of TREM2 in cancer. Cell.

[13] Jaitin DA (2019). Lipid-associated macrophages control metabolic homeostasis in a Trem2-dependent manner. Cell.

[14] Barrett LW, Fletcher S, Wilton SD (2012). Regulation of eukaryotic gene expression by the untranslated gene regions and other non-coding elements. Cell Mol. Life Sci..

[15] Iacono M, Mignone F, Pesole G (2005). uAUG and uORFs in human and rodent 5’untranslated mRNAs. Gene.

[16] Zhang H, Wang Y, Lu J (2019). Function and evolution of upstream ORFs in eukaryotes. Trends Biochem. Sci..

[17] Zhang H (2021). Determinants of genome-wide distribution and evolution of uORFs in eukaryotes. Nat. Commun..

[18] Michel AM (2014). GWIPS-viz: development of a ribo-seq genome browser. Nucleic Acids Res..

[19] Su X (2015). Interferon-γ regulates cellular metabolism and mRNA translation to potentiate macrophage activation. Nat. Immunol..

[20] Jang C, Lahens NF, Hogenesch JB, Sehgal A (2015). Ribosome profiling reveals an important role for translational control in circadian gene expression. Genome Res..

[21] Xu B, Gogol M, Gaudenz K, Gerton JL (2016). Improved transcription and translation with L-leucine stimulation of mTORC1 in Roberts syndrome. BMC Genomics.

[22] Ji Z, Song R, Huang H, Regev A, Struhl K (2016). Transcriptome-scale RNase-footprinting of RNA-protein complexes. Nat. Biotechnol..

[23] Gameiro PA, Struhl K (2018). Nutrient deprivation elicits a transcriptional and translational inflammatory response coupled to decreased protein synthesis. Cell Rep..

[24] Eichhorn SW (2014). mRNA destabilization is the dominant effect of mammalian MicroRNAs by the time substantial repression ensues. Mol. Cell..

[25] Reid DW, Xu D, Chen P, Yang H, Sun L (2017). Integrative analyses of translatome and transcriptome reveal important translational controls in brown and white adipose regulated by microRNAs. Sci. Rep..

[26] Cho J (2015). Multiple repressive mechanisms in the hippocampus during memory formation. Science.

[27] Hornstein N (2016). Ligation-free ribosome profiling of cell type-specific translation in the brain. Genome Biol..

[28] Fields AP (2015). A regression-based analysis of ribosome-profiling data reveals a conserved complexity to mammalian translation. Mol. Cell..

[29] Castelo-Szekely V, Arpat AB, Janich P, Gatfield D (2017). Translational contributions to tissue specificity in rhythmic and constitutive gene expression. Genome Biol..

[30] Alvarez-Dominguez JR, Zhang X, Hu W (2017). Widespread and dynamic translational control of red blood cell development. Blood.

[31] Fradejas-Villar N (2016). The RNA-binding protein Secisbp2 differentially modulates UGA codon reassignment and RNA decay. Nucleic Acids Res..

[32] Janich P, Arpat AB, Castelo-Szekely V, Lopes M, Gatfield D (2015). Ribosome profiling reveals the rhythmic liver translatome and circadian clock regulation by upstream open reading frames. Genome Res..

[33] Atger F (2015). Circadian and feeding rhythms differentially affect rhythmic mRNA transcription and translation in mouse liver. Proc. Natl Acad. Sci..

[34] Lee S (2012). Global mapping of translation initiation sites in mammalian cells at single-nucleotide resolution. Proc. Natl Acad. Sci..

[35] Hurt JA, Robertson AD, Burge CB (2013). Global analyses of UPF1 binding and function reveal expanded scope of nonsense-mediated mRNA decay. Genome Res..

[36] Laguesse S (2015). A dynamic unfolded protein response contributes to the control of cortical neurogenesis. Dev. Cell..

[37] Guo H, Ingolia NT, Weissman JS, Bartel DP (2010). Mammalian microRNAs predominantly act to decrease target mRNA levels. Nature.

[38] Castañeda J (2014). Reduced pachytene piRNAs and translation underlie spermiogenic arrest in Maelstrom mutant mice. EMBO J..

[39] Ansari SA (2022). Integrative analysis of macrophage ribo-Seq and RNA-Seq data define glucocorticoid receptor regulated inflammatory response genes into distinct regulatory classes. Comput. Struct. Biotechnol. J..

[40] Scheckel, C., Imeri, M., Schwarz, P. & Aguzzi, A. Ribosomal profiling during prion disease uncovers progressive translational derangement in glia but not in neurons. *Elife*. **9**, e62911 (2020). 10.7554/eLife.62911 PMC7527237 32960170

[41] Park JS (2015). Disease-associated mutations of TREM2 alter the processing of N-linked oligosaccharides in the golgi apparatus. Traffic.

[42] Yanaizu M, Washizu C, Nukina N, Satoh JI, Kino Y (2020). CELF2 regulates the species-specific alternative splicing of TREM2. Sci. Rep..

[43] Zhou Y (2020). Human and mouse single-nucleus transcriptomics reveal TREM2-dependent and TREM2-independent cellular responses in Alzheimer’s disease. Nat. Med..

[44] Eastman G, Sharlow ER, Lazo JS, Bloom GS, Sotelo-Silveira JR (2022). Transcriptome and translatome regulation of pathogenesis in alzheimer’s disease model mice. J. Alzheimers Dis..

[45] Gretzmeier C (2017). Degradation of protein translation machinery by amino acid starvation-induced macroautophagy. Autophagy.

[46] Jackson RJ, Hellen CUT, Pestova TV (2010). The mechanism of eukaryotic translation initiation and principles of its regulation. Nat. Rev. Mol. Cell Biol..

[47] Bendtsen JD, Nielsen H, von Heijne G, Brunak S (2004). Improved prediction of signal peptides: SignalP 3.0. J. Mol. Biol..

[48] Hiss JA (2008). Domain organization of long signal peptides of single-pass integral membrane proteins reveals multiple functional capacity. PLoS One.

[49] Rehm A, Stern P, Ploegh HL, Tortorella D (2001). Signal peptide cleavage of a type I membrane protein, HCMV US11, is dependent on its membrane anchor. EMBO J..

[50] Akari H (1996). In vitro immortalization of Old World monkey T lymphocytes with Herpesvirus saimiri: its susceptibility to infection with simian immunodeficiency viruses. Virology.

[51] Yanaizu M, Sakai K, Tosaki Y, Kino Y, Satoh JI (2018). Small nuclear RNA-mediated modulation of splicing reveals a therapeutic strategy for a TREM2 mutation and its post-transcriptional regulation. Sci. Rep..

[52] Satoh JI, Kino Y, Yanaizu M, Ishida T, Saito Y (2018). Microglia express gamma-interferon-inducible lysosomal thiol reductase in the brains of Alzheimer’s disease and Nasu-Hakola disease. Intractable Rare Dis. Res..

[53] Koike, S. et al. Accumulation of carbonyl proteins in the brain of mouse model for methylglyoxal detoxification deficits. *Antioxidants (Basel)*. **10**, 574 (2021). 10.3390/antiox10040574 PMC8068291 33917901

