# Supplementary material for: Translational regulation and protein-coding capacity of the 5′ untranslated region of human TREM2
Source: Commun Biol. 2023 Jun 8;6:616. doi: 10.1038/s42003-023-04998-6 (PMC10250343; doi:10.1038/s42003-023-04998-6)
Supplement: Supplementary file 1 — Supplementary Information [file 42003_2023_4998_MOESM1_ESM.pdf]

Supplementary Information

Translational regulation and protein coding capacity of the 5′ untranslated region of human *TREM2*

Motoaki Yanaizu[1,2], Haruka Adachi[1], Makoto Araki[3], Kenji Kontani[3], Yoshihiro Kino[1,2,*]

[1]Department of Bioinformatics and Molecular Neuropathology, Meiji Pharmaceutical University, 2-522-1, Noshio, Kiyose-shi, Tokyo 204-8588, Japan

[2]Department of RNA Pathobiology and Therapeutics, Meiji Pharmaceutical University, 2-522-1, Noshio, Kiyose-shi, Tokyo 204-8588, Japan

[3]Department of Biochemistry, Meiji Pharmaceutical University, 2-522-1, Noshio, Kiyose-shi, Tokyo 204-8588, Japan

[*]Corresponding author: Tel.: +81-42-495-8679, E-mail: kino@my-pharm.ac.jp

E-mail addresses:

Motoaki Yanaizu: m-yana@my-pharm.ac.jp

Haruka Adachi: m186202@alm.my-pharm.ac.jp

Makoto Araki: maraki@my-pharm.ac.jp

Kenji Kontani: kontani@my-pharm.ac.jp

Yoshihiro Kino: kino@my-pharm.ac.jp

Supplementary Figure 2a	GEO ID	BioProject ID	Sample source	SRP
**Human TREM2**	GSE66810	PRJNA278006	Control or IFN-γ–treated human primary macrophages	SRP056098
	GSE56924	PRJNA244941	U2OS cell transfected with control siRNA or siBMAL1	SRP041298
	GSE64962	PRJNA272597	human fibroblast cell lines treated with l-leucine or d-leucine	SRP052229
	GSE65885	PRJNA275305	a Src-inducible mammary epithelial model and a Ras-dependent fibroblast model	SRP054971
	GSE114794	PRJNA472636	MCF10A-ER-Src cells incubated without specific amino acid DMEM	SRP148767
**Mouse Trem2**	GSE60426	PRJNA258219	85 samples from a variety of cell types and species	SRP045501
	GSE89108	PRJNA350300	interscapular brown fat, epididymal white fat,<br>in vitro cultured white and brown preadipocytes and differentiated adipocytes	SRP091987
	GSE72064	PRJNA292861	mouse hippocampi tissues after contextual fear conditioning<br>and to mouse hippocampal primary cultures	SRP062407
	GSE78163	PRJNA312799	The forebrain of Camk2a-cre mice	SRP070655
	GSE74139	PRJNA299160	mouse BMDCs treated with LPS, harringtonine, lactimidomycin	SRP064972
	GSE81283	PRJNA321185	mouse livers and kidneys	SRP074765
	GSE83823	PRJNA327113	primary erythroid progenitors isolated from E14.5 mouse fetal livers	SRP077522
	GSE84112	PRJNA328042	Livers from WT mice and Secisbp2 or Sec-tRNA (Trsp)-depleted mice	SRP078005
	GSE67305	PRJNA279465	Livers isolated from mice	SRP056576
	GSE73553	PRJNA297288	mouse liver isolated from WT mice and Bmal$^{-/-}$ mice	SRP064304
	SRA056377	PRJNA171327 •	mouse embryonic fibroblast treated with lactimidomycin, harringtonine, cycloheximide, DMSO	SRA056377
	GSE41785	PRJNA178171	Upf1-depleted mouse embryonic stem cells	SRP016625
	GSE74683	PRJNA301154	forebrain from E14.5 mouse embryos (wild type and Elp3 knockout)	SRP065804
	GSE22004	PRJNA127467	mir-223 knockout cultured neutrophils and wild-type cultured neutrophils	SRP003554
	GSE50983	PRJNA219480	Testes isolated from P21 Mael129$^{+/+}$ and from P21 Mael129$^{-/-}$ mice	SRP030014

Supplementary Figure 2b	GEO ID	BioProject ID	Sample source	SRP
**Human TREM2**	GSE208041	PRJNA858047	THP-1 treated with 10 ng ml^{-1} PMA 3 h with vehicle (0.1% EtOH)	SRX16141475, SRX16141476, SRX16141478, SRX16141479
			THP-1 treated with 10 ng ml^{-1} PMA 3 h with 100 ng ml^{-1} LPS	SRX16141481, SRX16141483, SRX16141484
			THP-1 treated with 10 ng ml^{-1} PMA 3 h with 100 ng ml^{-1} LPS and 1mM Dexamethasone	SRX16141485, SRX16141487, SRX16141489
**Mouse Trem2**	GSE149805	PRJNA630198	mice (Cx3cr1^{Cre}/Rpl10a: GFP), hemisphere, no inoculation	SRX8245155, SRX8245156, SRX8245157
			mice (Cx3cr1^{Cre}/Rpl10a: GFP), hemisphere, 8 wk post prion inoculation	SRX8245158, SRX8245159, SRX8245160
			mice (Cx3cr1^{Cre}/Rpl10a: GFP), hemisphere, no inoculation	SRX8245113, SRX8245114, SRX8245133
			mice (Cx3cr1^{Cre}/Rpl10a: GFP), hemisphere, 16 wk post prion inoculation	SRX8245134, SRX8245135, SRX8245136
			mice (Cx3cr1^{Cre}/Rpl10a: GFP), hemisphere, no inoculation	SRX8245137, SRX8245138, SRX8245139
			mice (Cx3cr1^{Cre}/Rpl10a: GFP), hemisphere, 24 wk post prion inoculation	SRX8245140, SRX8245141, SRX8245142

Supplementary Table 1. List of Ribo-seq datasets.

	name	sequence (5' to 3')
chimeric 5'-UTR (chimpanzee)	BamHI-chimpTrem2-5'-UTR-Fw	AAAAGGATCCATGCCTGATCCTCTCTTTTCTGCAG
	ChimpTrem2-5'-UTR-Fw	AAGGGTGGCATGGAGCCTCTCCGGCTGCTC
	ChimpTrem2-5'-UTR-Rv	AGGCTCCATGCCACCCTTCCCCAGCCAAGG
	XbaI-hTREM2-Rv	AAAATCTAGATCACGTGTCTCTCAGCCCTGGCAGA
chimeric 5'-UTR (marmoset)	BamHI-MarmoTrem2-5'-UTR-Fw	AAAAGGATCCATGACTGATCCTCTCTTTTCTGCAG
	MarmoTrem2-5'-UTR-Fw	AAGGGTGGCATGGAGCCTCTCCGGCTGCTC
	MarmoTrem2-5'-UTR-Rv	AGGCTCCATGCCACCCTTTCCCAGCCAAGG
	XbaI-hTREM2-Rv	AAAATCTAGATCACGTGTCTCTCAGCCCTGGCAGA
chimeric 5'-UTR (mouse)	BamHI-mTrem2-5'-UTR-Fw	AAAAGGATCCGGCTTGGTCATCTCTTTTCTGCACT
	mTrem2-5'-UTR-Rv	GGCTCCATGGCACCTTTCCTTTGCCAGCAGCCAGC
	mTrem2-5'-UTR-Fw	AAGGTGCCATGGAGCCTCTCCGGCTGCTCATCTTA
	XbaI-hTREM2-Rv	AAAATCTAGATCACGTGTCTCTCAGCCCTGGCAGA
A series of fl-TREM2 minigenes containing 5'-UTR	CMV-Fw2	TCCAAGTCTCCACCCCA
	TREM2-ex1-mid-Fw	ATGGAGCCTCTCCGGCTGCTCATCTTACTCTTTGT
	TREM2-ex1-mid-Rv	ACAAAGAGTAAGATGAGCAGCCGGAGAGGCTCCAT
	TREM2-check-Rv3	CCTCCAGAATAGACCCAGAGAA
	NheI-TREM2-uATG-Fw	AAAAGCTAGCTGACATGCCTGATCCTCTCTTTTCT
	NheI-TREM2-MuATG-Fw	AAAAGCTAGCTGACGTGCCTGATCCTCTCTTTTCT
	TREM2-int1-HindIII-Rv	TTATAATTTCAGAGGCTATTACCAGTGCCA
fl-TREM2 minigene 5'-UTR-F6X	CMV-Fw2	TCCAAGTCTCCACCCCA
	uTREM2-F6X-Fw	GATCCTCTCTAGTCTGCAGTTCAAGGGAAAGACGA
	uTREM2-F6X-Rv	AGACTAGAGAGGATCAGGCATGTCAGCTAGCCAGC
	TREM2-int1-HindIII-Rv	TTATAATTTCAGAGGCTATTACCAGTGCCA
fl-TREM2 minigene 5'-UTR-C23X	CMV-Fw2	TCCAAGTCTCCACCCCA
	TREM2-C23X-Fw	CACTCTGCTTCTGACCTTGGCTGGGGAAGGGTGGC
	TREM2-C23X-Rv	TCCCCAGCCAAGGTCAGAAGCAGAGTGCCTTGTGC
	TREM2-int1-HindIII-Rv	TTATAATTTCAGAGGCTATTACCAGTGCCA
fl-TREM2 minigene 5'-UTR-FS	CMV-Fw2	TCCAAGTCTCCACCCCA
	T2-5'-UTR-FS-Fw	GACGAGATCATTGCACAAGGCACTCTGCTTCTGCC
	T2-5'-UTR-FS-Rv	CCTTGTGCAATGATCTCGTCTTTCCCTTGAACTGC
	TREM2-int1-HindIII-Rv	TTATAATTTCAGAGGCTATTACCAGTGCCA
Quantitative PCR	RT-TREM2-Fw2	TCTGAGAGCTTCGAGGATGC
	RT-TREM2-Rv2	GGGGATTTCTCCTTCCAAGA
	RT-ACTB-Fw	GGACCTGACTGACTACCTCAT
	RT-ACTB-Rv	CGTAGCACAGCTTCTCCTTAAT

Supplementary Table 2. List of primer sequences.

		source	identifier	concentration
primary antibodies	Goat anti-TREM2	R&D	AF1828	1/600
	Rabbit anti-TREM2	Cell Signaling Technology	D8I4C	1/1000
	Rabbit anti-SQSTM1	proteintech	18420-1-AP	1/2000
	Goat anti-HSP60	Everest biotech	EB12834	1/5000
	Rabbit anti-APP	abcam	ab32136	1/5000
	Mouse anti-GAPDH	MBL	M171-3	1/1000
	Rabbit anti-LC3	MBL	PM036	1/1000
	Rabbit anti-phospho-eIF2α (pSer51)	Sigma	SAB4504388	1/500
	Rabbit anti-Ubiquitin	Dako	Z0458	1/2000
secondary antibodies	Donkey anti-goat IgG (HRP)	abcam	ab97120	1/5000
	Goat anti-mouse IgG (HRP)	abcam	ab97040	1/5000
	Goat anti-rabbit IgG (HRP)	Jackson ImmunoResearch	111-035-144	1/5000
	Mouse anti-rabbit IgG (HRP)	ROCKLAND	eB182	1/1000
	Alexa Fluor 488 goat anti-rabbit IgG (H+L)	invitrogen	A11034	1/2000
	Alexa Fluor 568 donkey anti-rabbit IgG (H+L)	invitrogen	A10042	1/2000

Supplementary Table 3. List of antibodies.

Supplementary Figure 1

Human	AUGCCUGAUCCUCUCUUUUCUGCAGUUCAAGGGAAAGACGAGAUCUUGCACAAGGCACUCUGCUUCUGCCCUUGGCUGGGGAAGGGUGGCAUG
Chimpanzee	AUGCCUGAUCCUCUCUUUUCUGCAGUUCAAGGGAAAGACGAGAUCUUGCACAAGGCACUCUGCAUCUGCCCUUGGCUGGGGAAGGGUGGCAUG
Bonobo	AUGCCUGAUCCUCUCUUUUCUGCAGUUCAAGGGAAAGACGAGAUCUUGCACAAGGCACUCUGCAUCUGCCCUUGGCUGGGGAAGGGUGGCAUG
Gorilla	AUGCCUGAUCCUCUCUUUUCUGCAGUUCAAGGGAAAGACGAGAUCUUGCACAAGGCACUCUGCAUCUGCCCUUGGCUGGGGAAGGGUGGCAUG
Orangutan	AUGCCUGAUCCUCUCUUUUCUGCAGUUCAAGGGAAAGACGAGAUCUUGCACAAGGCACUCUGCAUCUGCCCUUGGCUGGGGAAGGGUGGCAUG
Gibbon	AUGCCUGAUCCUCUCUUUUCUGCAGUUCAAGGGAAAGAUGAGAUCUUGCACAAGGCACUCUGCAUCUGCCCUUGGCUGGGGAAGGGUGGCAUG
Golden snub-nosed monkey	AUGCCUGAUCCUCUCUUUUCUGCAGUUCAAGGGAAAGACAAGAUCUUGCACAAGGCACUCUGCAUCUGCCCUUGGCCAGGGAAGGGUGGCAUG
Vervet-AGM	AUGCCUGAUCCUCUCUUUUCUGCAGUUCAAGGGAAAGACAAGAUCUUGCACAAGGCACUCUGCAUCUGCCCUUGGCCAGGGAAGGGUGGCAUG
Macaque	AUGCCUGAUCCUCUCUUUUCUGCAGUUCAAGGGAAAGACAAGAUCUUGCACAAGGCACUCUGCAUCUGCCCUUGGCCAGGGAAGGGUGGCAUG
Crab-eating macaque	AUGCCUGAUCCUCUCUUUUCUGCAGUUCAAGGGAAAGACAAGAUCUUGCACAAGGCACUCUGCAUCUGCCCUUGGCCAGGGAAGGGUGGCAUG
Drill	AUGCCUGAUCCUCUCUUCUCUGCAGUUCAAGGGAAAGACAAGAUCUUGCACAAGGCACUCUGCAUCUGCCCUUGGCCAGGGAAGGGUGGCAUG
Sooty mangabey	AUGCCUGAUCCUCUCUUUUCUGCAGUUCAAGGGAAAGACAAGAUCUUGCACAAGGCACUCUGCAUCUGCCCUUGGCCAGGGAAGGGUGGCAUG
Ma’s night monkey	AUGCCUGAUCCUCUCUUUUCUGCAGUUCAAGGGAAAGACGAGAUCUUGCACAAGGCACUCUGCAUCUUCCCUUGGCUGGGAAAGGGUGGCAUG
Capuchin	AUGCCUGAUCCUCUCUUUUCUGCAGUUCAAGGGAAAGACGAGAUCUUGCACAAGGCACUCUGCAUCUUCCCUUGGCUGAGAAAGGGUGGCAUG
Marmoset	AUGACUGAUCCUCUCUUUUCUGCAGUUCAAGGGAAAGACGAGAUCUUGCACAAGGCACUCUGCAUCUUCCCUUGGCUGGGAAAGGGUGGCAUG
Coquerel’s sifaka	AUGUGCCUGAUCCUCGCUUUUGUGCAGUCCGAGGGAAAGAGGAGAUCUUGCACAAGGCGCCCUGUGUCUGCCCUCAGUGGGGAAGGCGGCAUG
Black snub-nosed monkey	UGACAUCCAAAAGGAGAAAACCACUUUCAAGGGAAAGACAAGAUCUUGCACAAGGCACUCUGCAUCUGCCCUUGGCCAGGGAAGAGUGGCAUG
Pig-tailed macaque	UGACGUCCAAAAGGAGAAAACCACUUUCAAGGGAAAGACAAGAUCUUGCACAAGGCACUCUGCAUCUGCCCUUGGCCAGGGAAGGGUGGCAUG
Olive baboon	UGACGUCCAAAAGGAGAAAACCACUUUCAAGGGAAAGACAAGAUCUUGCACAAGGCACUCUGCAUCUGCCCUUGGCCAGGGAAGGGUGGCAUG
Greater bamboo lemur	UGAUGUGCCUGAUCCCCGCUUUAGUGCAGUUCGAGGAAAGAGGAAUCUUGCACAAGGCGCCCUGUGUCUGCCCUCAGCGGGGAAGCUGGCAUG
Bolivian squirrel monkey	ACAUGACUGAUCCUCUCUUCUGCAGUUCAAGGGAAAGACAAGAUCUUGCACAAGACAGUCUGCAUCUUCCCUUAGCUGGGACAGGGUGGUAUG
Bushbaby	ACUUGCCUGAUCCUCUGUUUUGUGCAGUUCAAGGGAAAGACAAGAUCUUGCACAAGGCACCCUGUGUCUGCCCUCAGUGGGAAAGGUGGCAUG
Mouse lemur	CCUGGUGUGCCUGACCCUCGCCUUGUGCCCUUCAGGGAAGGAGGGAUCUUGCACAAGGCGCCCUGUGUCUGCCCUCAGCGGGAAGGCGGCAUG
Tarsier	CGUGCCUGAUCCUCUCUUUUCUGCAGUUUAAGGGAAAGACGAAGUCUUGCACAAGGCACUCUGUGUCUGCUCUUGGCAAGGAAGGGUGGCAUG

Supplementary Figure 1. Sequence alignment of primate TREM2.

The RNA sequence 90 bases upstream of downstream AUG (dAUG) in primate TREM2 was registered in the Ensembl Genome Browser. The upstream AUG (uAUG) is highlighted in green.

Supplementary Figure 2

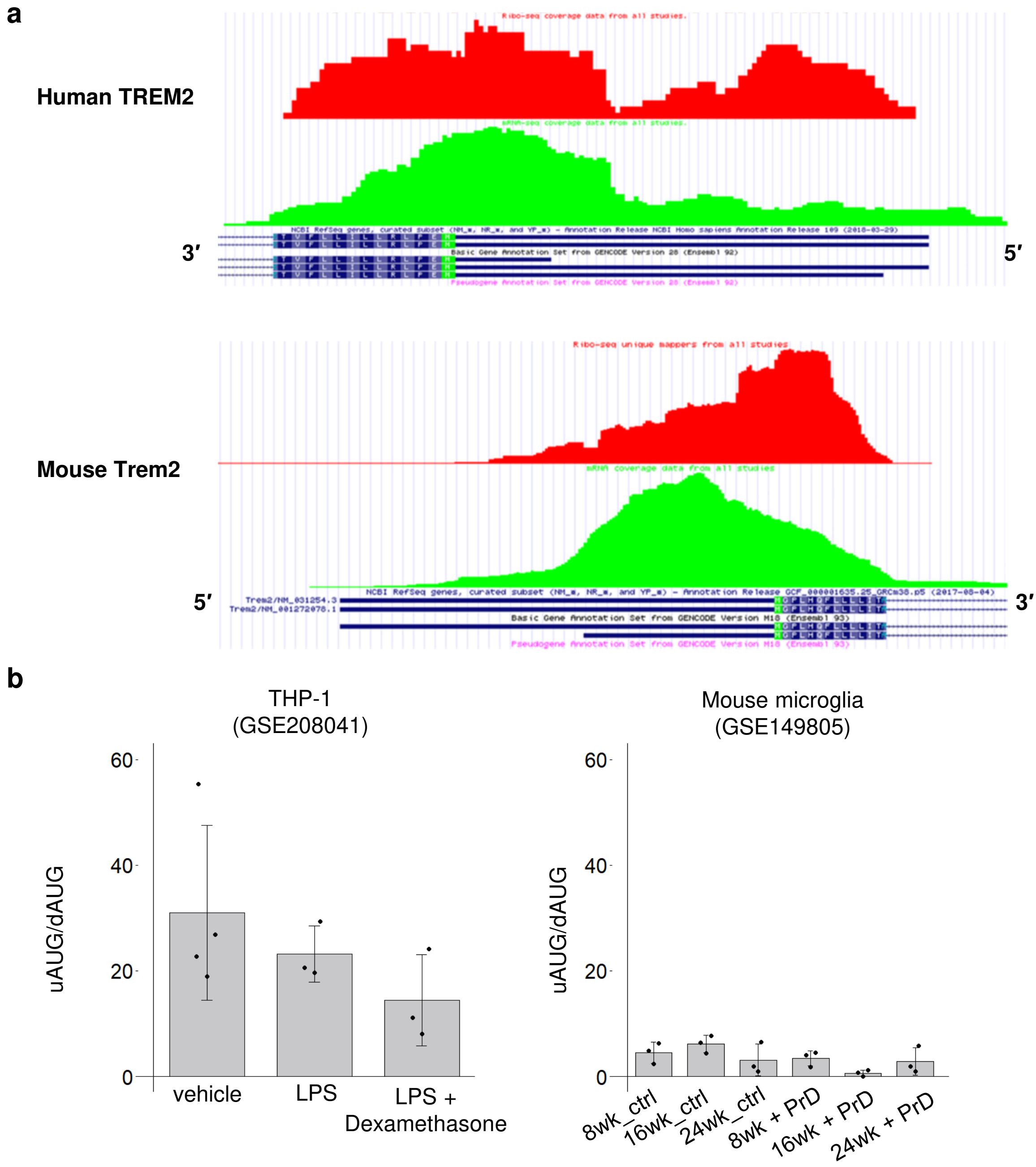

Supplementary Figure 2. Ribosome profiling of the of human and mouse TREM2.

a The profile of ribosome-bound TREM2 mRNA was obtained based on the 5′-UTR (untranslated region) from GWIPS-viz (human TREM2: GSE66810, GSE56924, GSE64962, GSE65885, GSE114794; and mouse Trem2: GSE60426, GSE89108, GSE72064, GSE78163, GSE74139, GSE81283, GSE83823, GSE84112, GSE67305, GSE73553, SRA056377, GSE41785, GSE74683, GSE22004, GSE50983). Red and green peaks represent the ribo-seq and mRNA-seq signals, respectively. **b** Initiation codon usage of TREM2 and Trem2. Relative usage of uAUG was determined as the ratio of read counts in two regions (45 nucleotides from uAUG and 45 nucleotides from dAUG) in a ribo-seq dataset (GSE208041). Similarly, mouse Trem2 was analyzed was using the corresponding regions (GSE149805). Information of ribo-seq datasets is summarized in Supplementary Table 3. Error bars represent the mean ± SD (n = 4 for vehicle-treated THP-1, and n = 3 for all other samples). PrD: prion disease.

Supplementary Figure 3

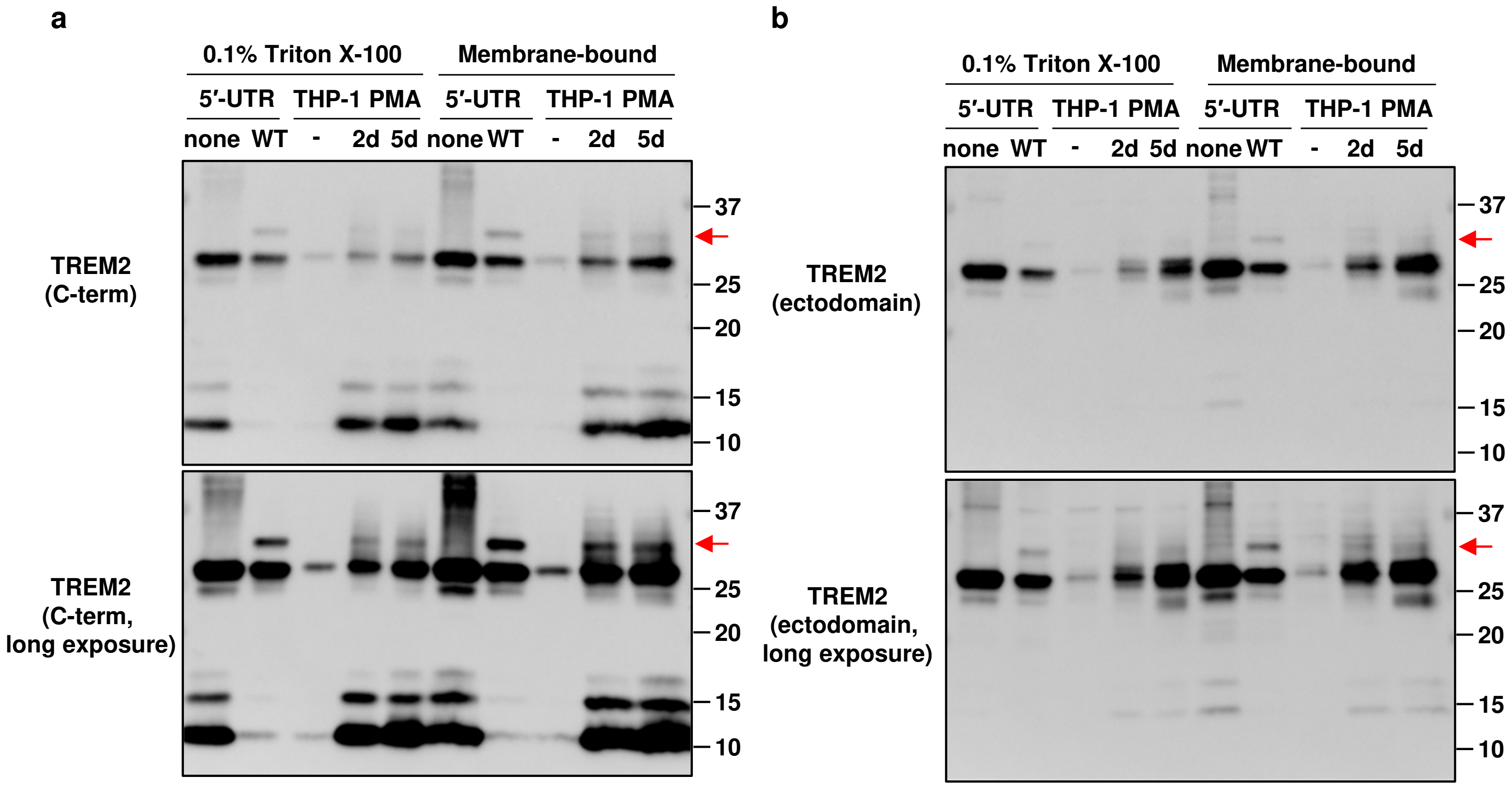

Supplementary Figure 3. Cell fractionation facilitates the detection of uTREM2 (TREM2 protein isoform starting from upstream AUG).

a, b The total cell lysate was solubilized in a buffer containing 0.1% Triton X-100. After centrifugation, the supernatant was used for western blot analysis. Equal amounts of proteins were subjected to western blot analysis. THP-1 cells were used as a cell type expressing endogenous human TREM2. Red arrows indicate the protein bands of uTREM2.

Supplementary Figure 4

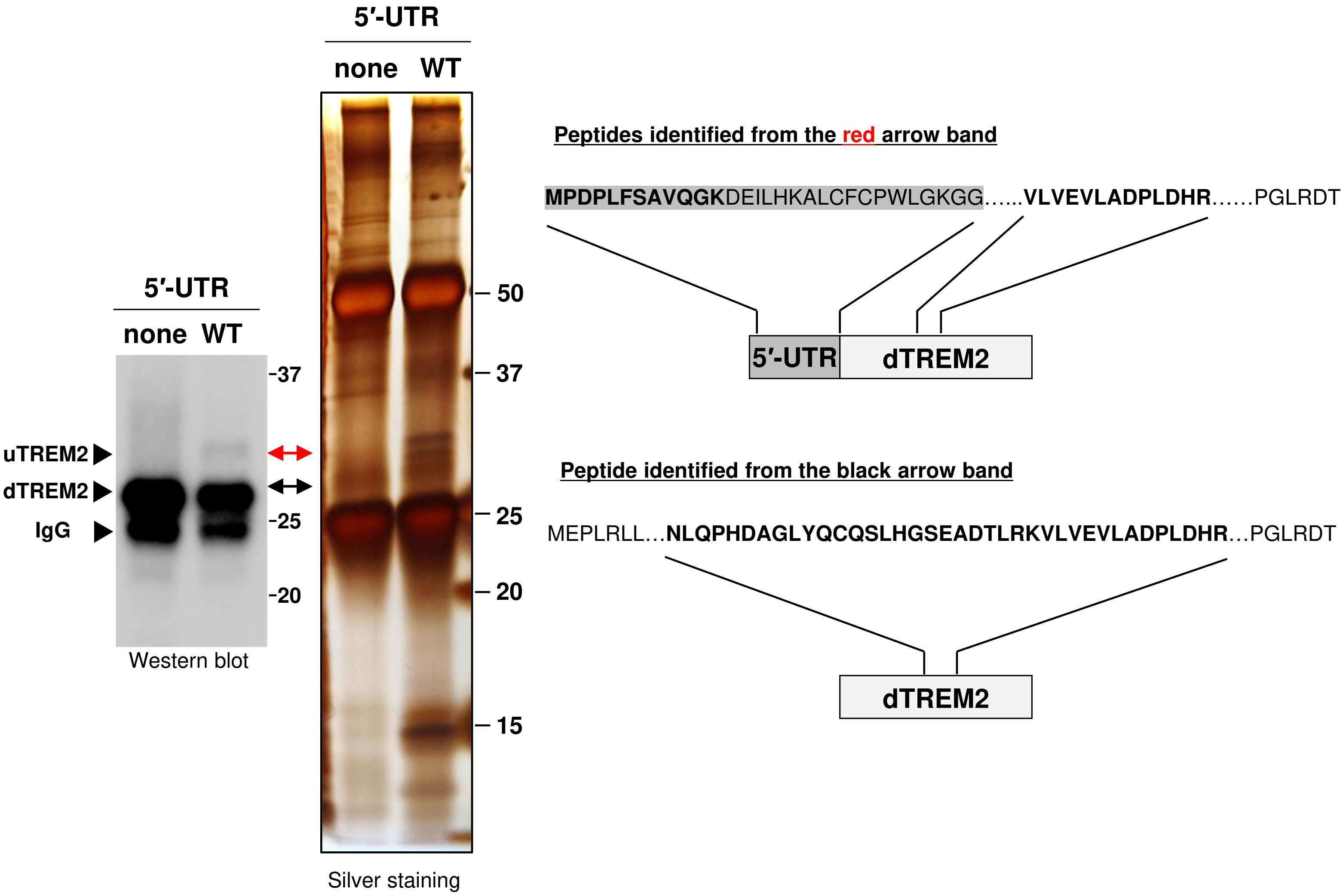

Supplementary Figure 4. LC-MS/MS analysis of excised uTREM2 protein band.

The 5′-UTR-none and WT cell lines were subjected to SDS-PAGE and silver staining following immunoprecipitation. The black and red arrows indicate the excised band from the SDS-PAGE gel. The extended N-terminal amino acid sequence specific to uTREM2 is highlighted in gray. The identified peptides are highlighted in bold.

Supplementary Figure 5

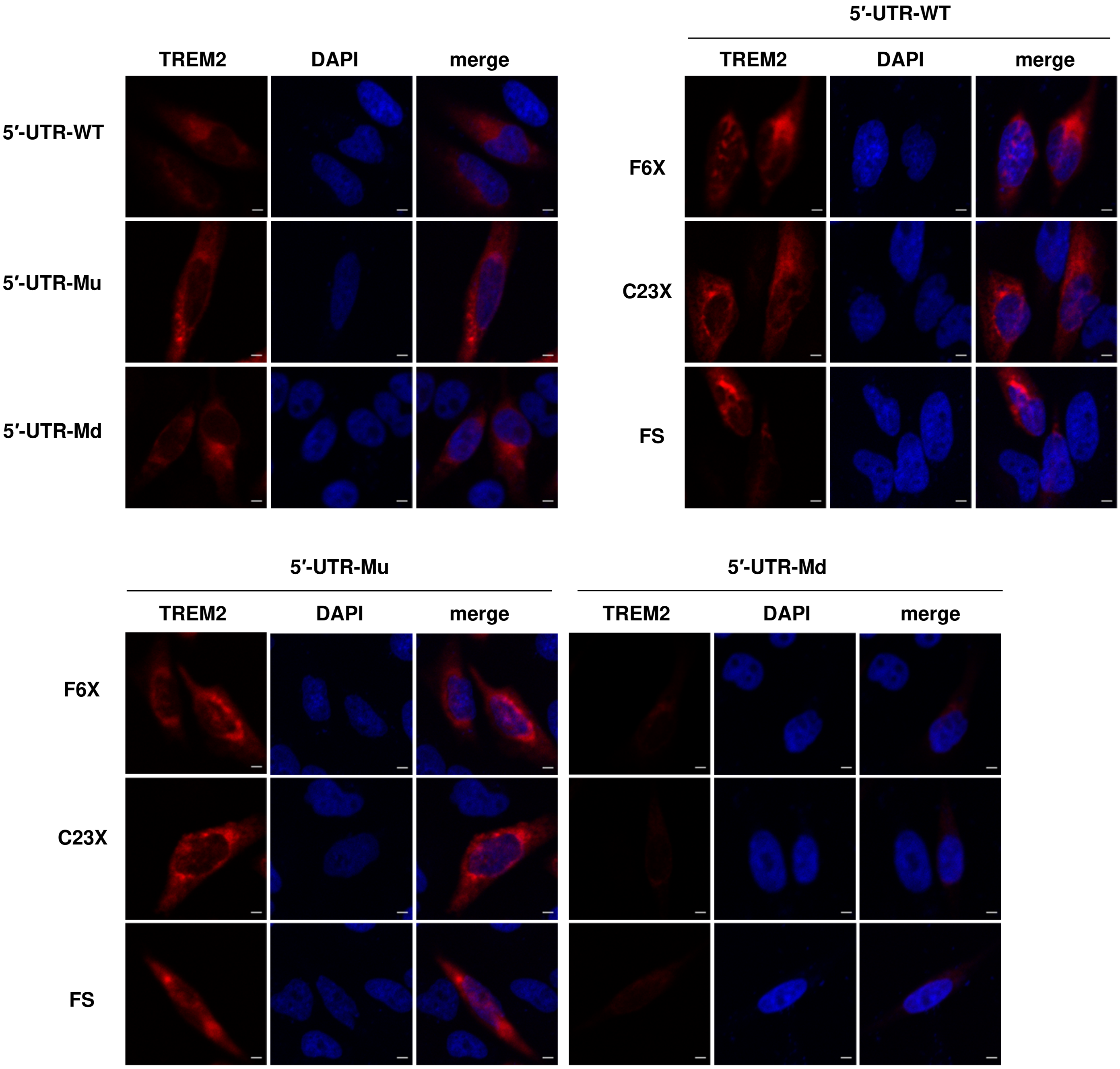

Supplementary Figure 5. Localization of TREM2 mutants expressed in HeLa cells

TREM2 minigenes were transfected into HeLa cells and immunostained with anti-TREM2 antibody. Scale bar represents 5 µm.

Supplementary Figure 6

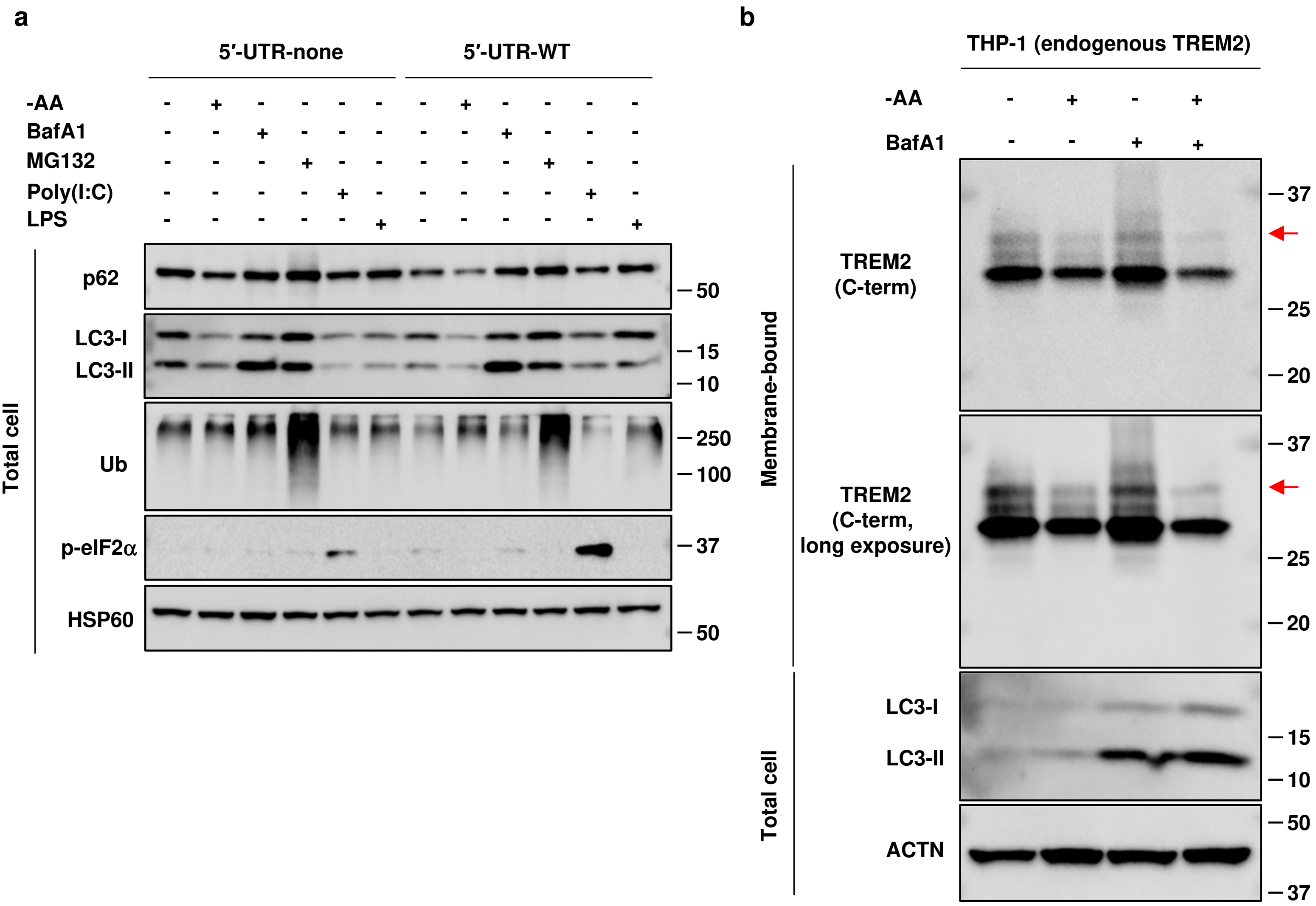

Supplementary Figure 6. Effects of cellular stresses on the Flp-In cell lines (related to Figure 5).

a Total cell lysates were analyzed by western blotting using the indicated antibodies to confirm the effect of cellular stress. **b** THP-1 cells were exposed to amino acid starvation with or without BafA1 treatment and were analyzed by western blotting. Red arrows indicate the protein band of uTREM2.

Supplementary Figure 7

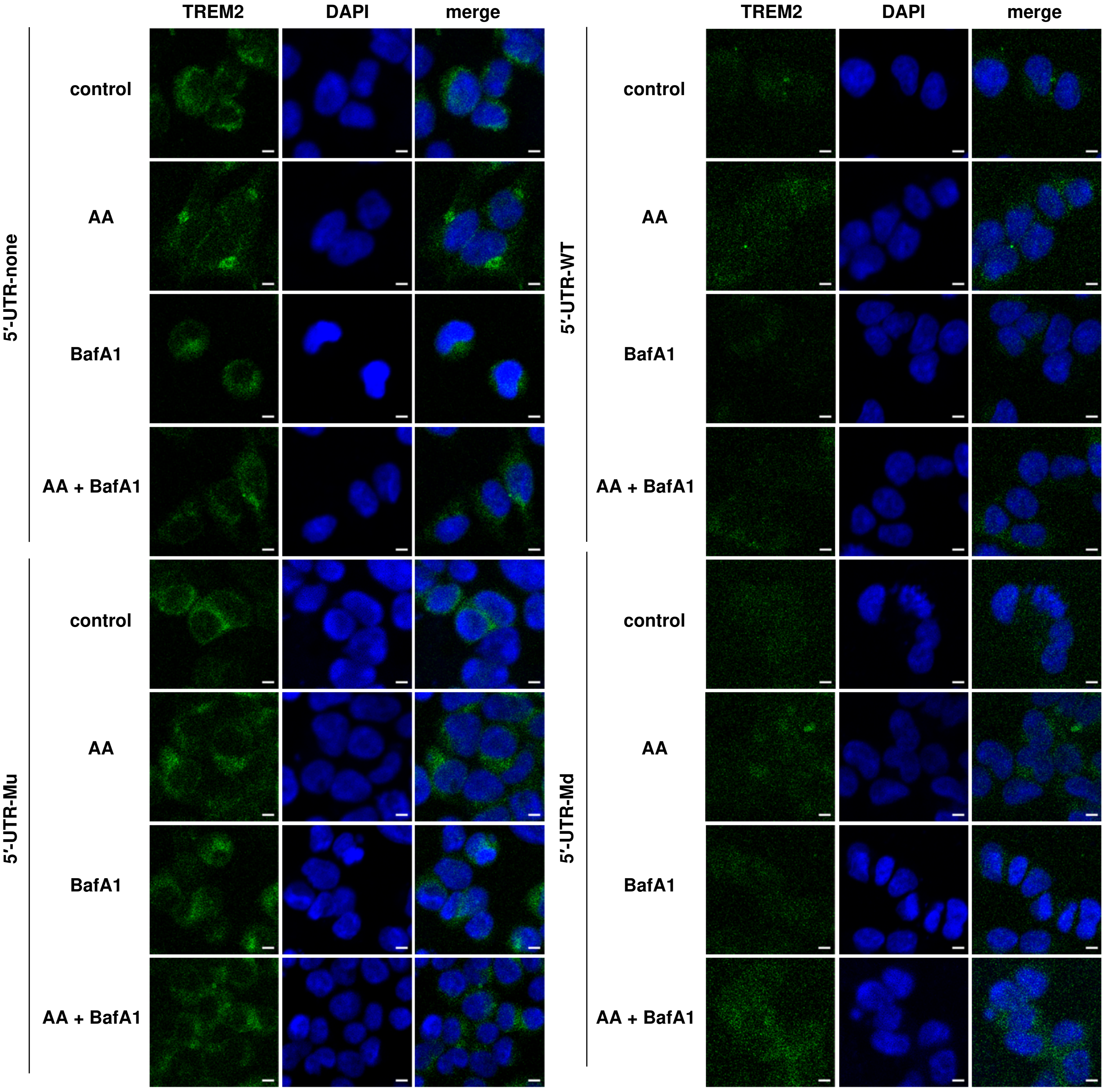

Supplementary Figure 7. Immunofluorescence analysis of stable cell lines expressing 5′-UTR-containing TREM2 minigene under stress conditions.

TREM2 protein expressed in the stable cell lines was labeled with anti-TREM2 C-terminal antibody. Scale bars represent 5 μm.

Supplementary Figure 8

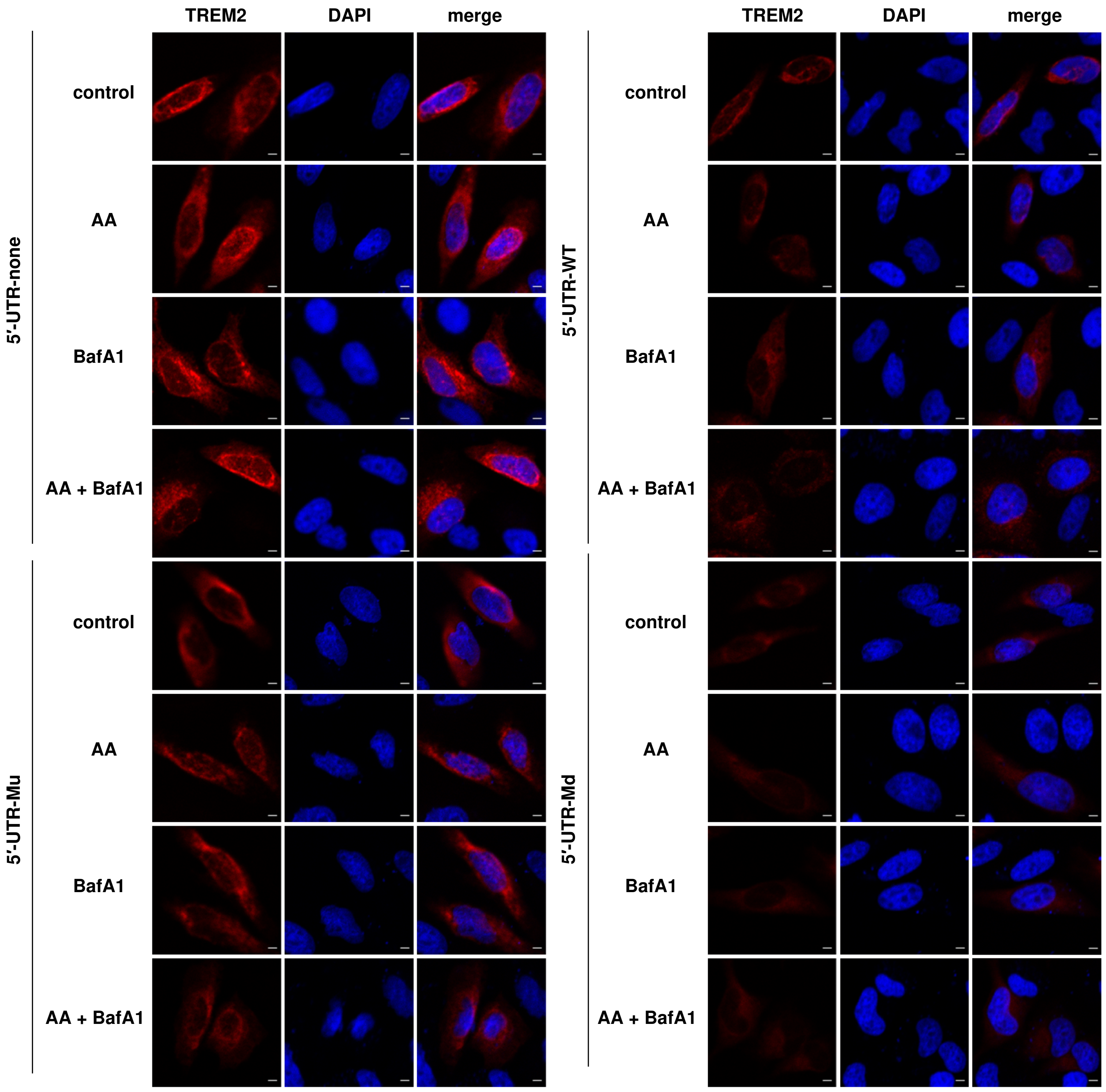

Supplementary Figure 8. Immunofluorescence analysis of cells transiently expressing the 5′-UTR-containing TREM2 minigenes.

HeLa cells transfected with the 5′-UTR-containing full-length TREM2 minigene in different conditions were labeled with anti-TREM2 C-terminal antibody. Scale bars represent 5 µm.

Supplementary Figure 9

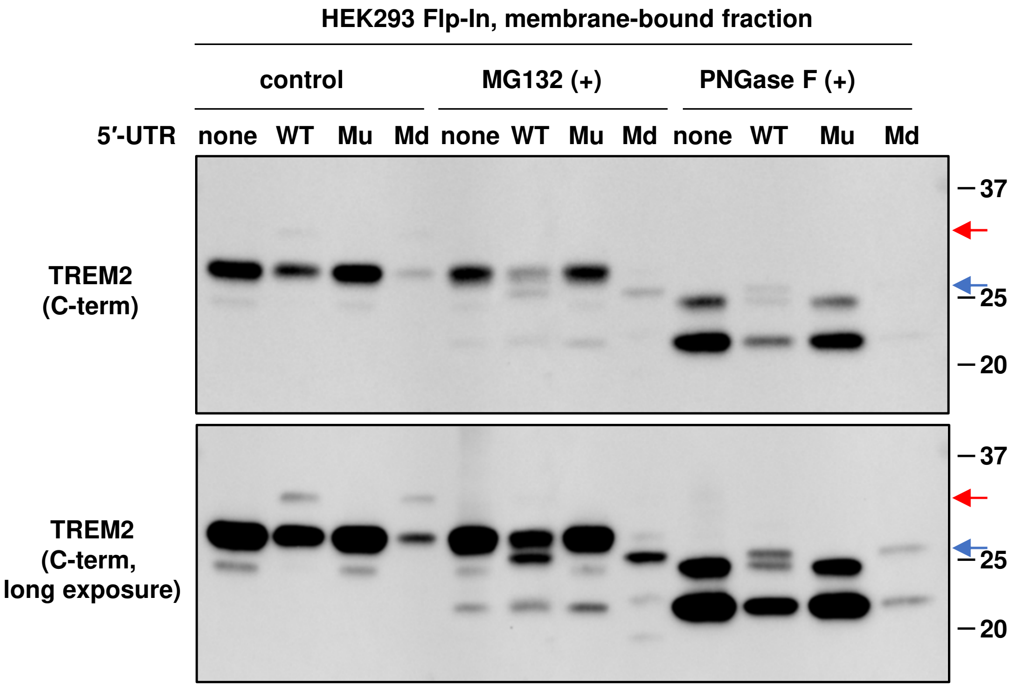

Supplementary Figure 9. Comparison of proteasome-sensitive uTREM2 and deglycosylated uTREM2.

Membrane-bound protein fractions of stable cell lines treated with MG132 or PNGase F were subjected to western blot analysis. Red and blue arrows indicate the protein band of uTREM2 and deglycosylated uTREM2, respectively.

Supplementary Figure 10

Fig.1c

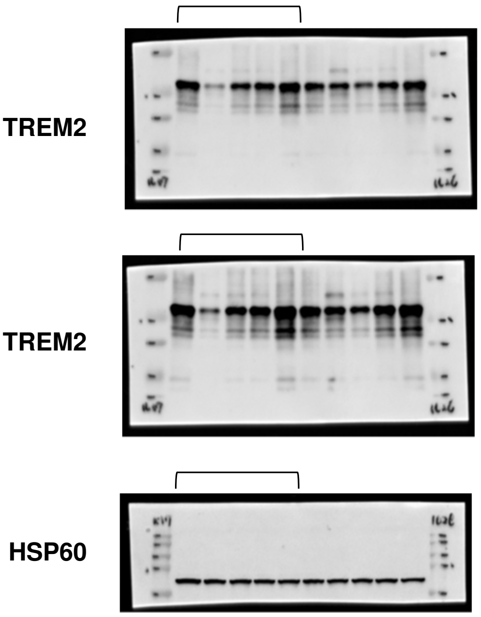

Fig.2b

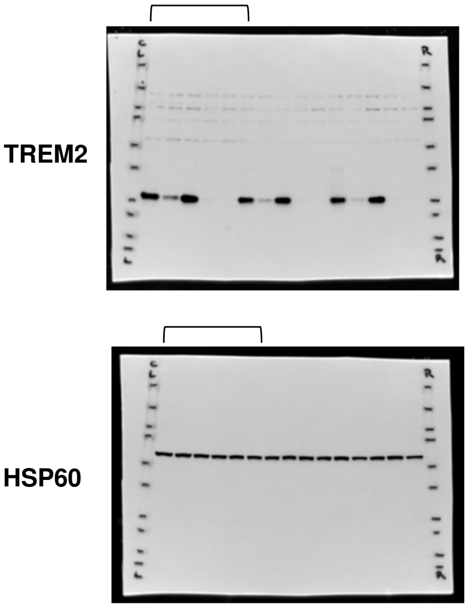

Fig.3a

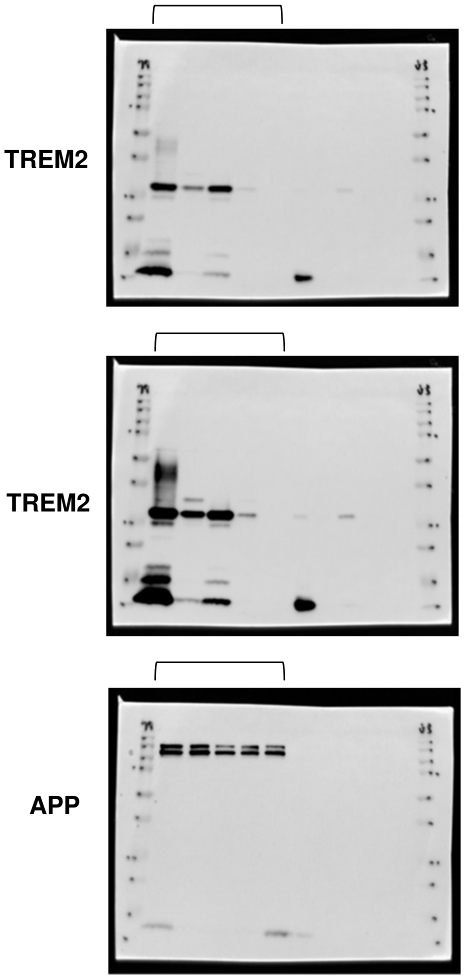

Fig.3c

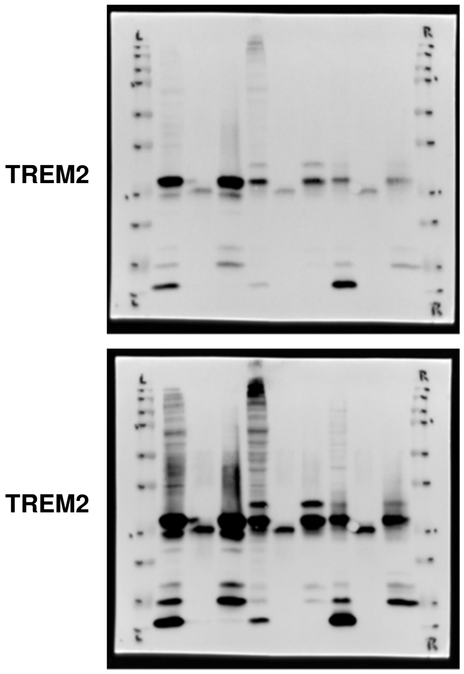

Fig.3d

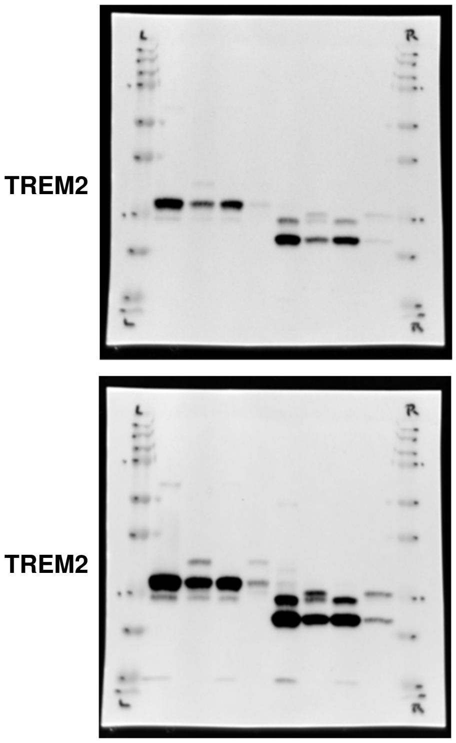

Fig.3e

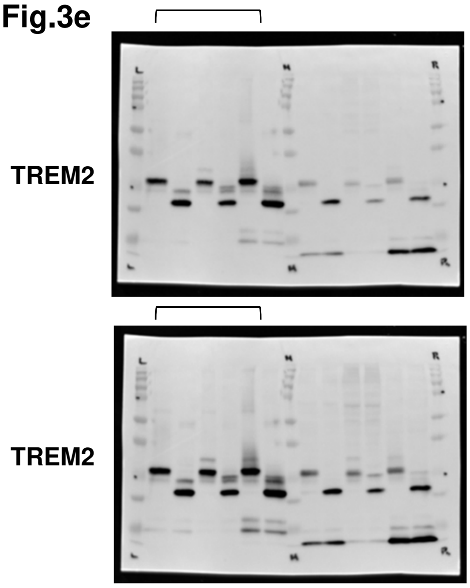

Supplementary Figure 10. Original uncropped images for figures.

Supplementary Figure 10 (continued)

Fig. 4b

TREM2

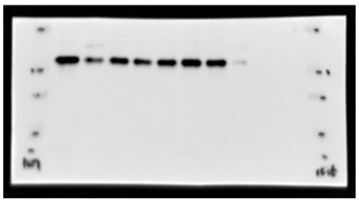

TREM2

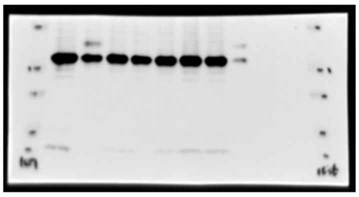

APP

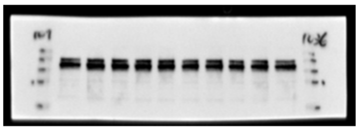

Fig. 4d

TREM2

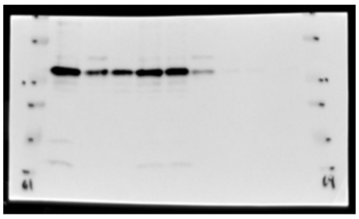

TREM2

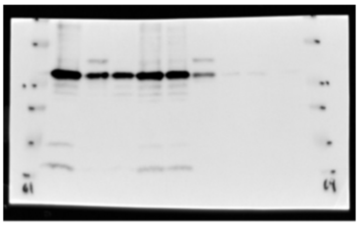

APP

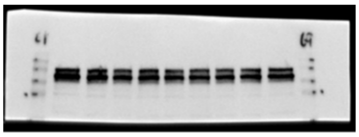

Fig. 5a

TREM2

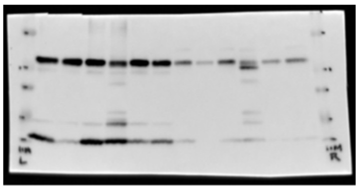

TREM2

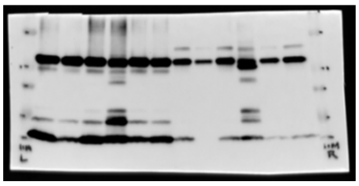

Fig. 5e

TREM2

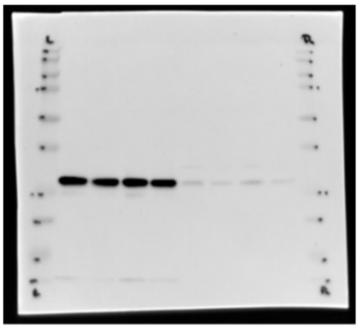

TREM2

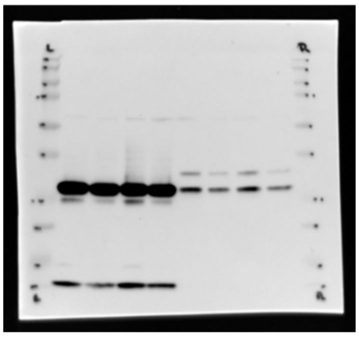

p62

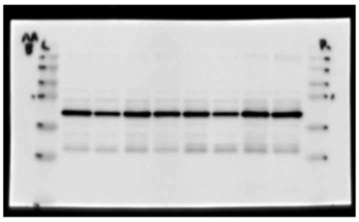

LC3

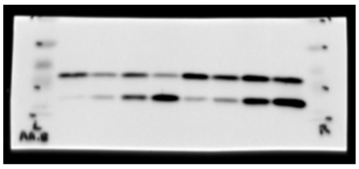

HSP60

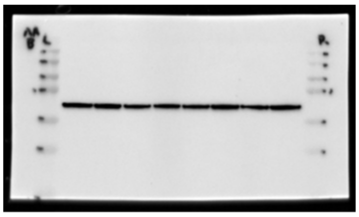

Fig. 5d

TREM2

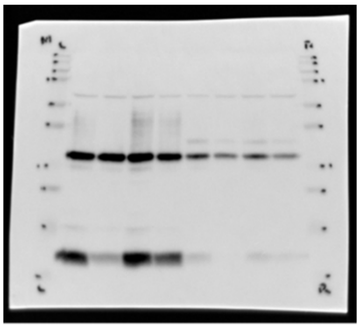

TREM2

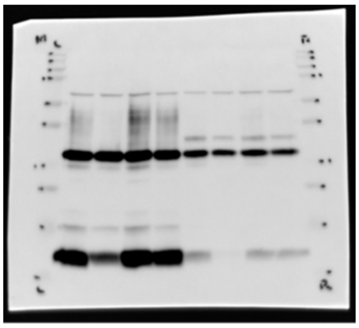

Fig. 5d

p62

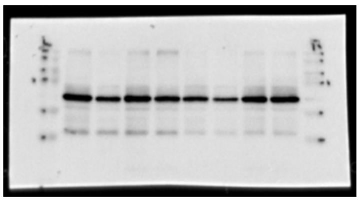

LC3

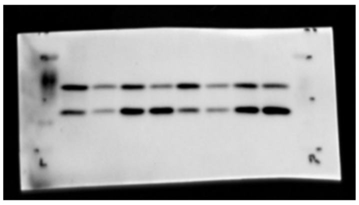

HSP60

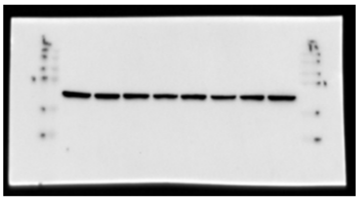

Supplementary Figure 10. Original uncropped images for figures.

Supplementary Figure 10 (continued)

Fig. 6a

TREM2

TREM2

HSP60

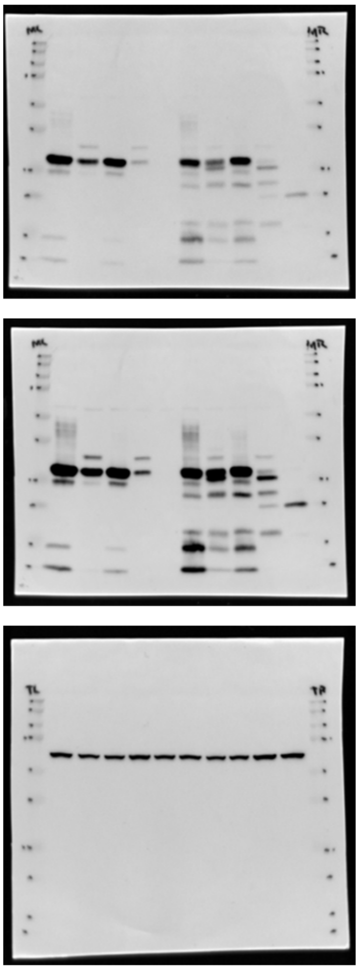

Fig. 6b

TREM2

TREM2

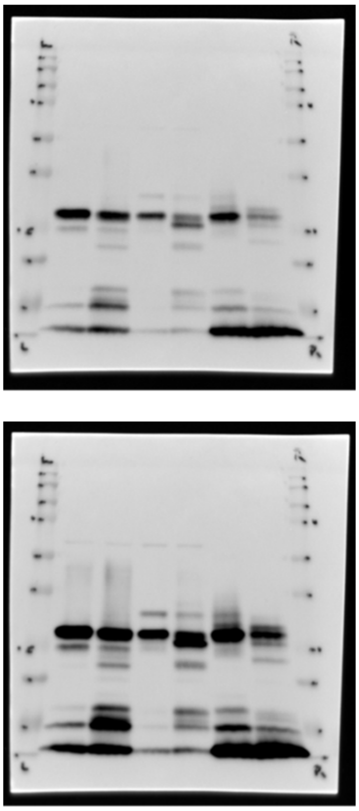

Ub

GAPDH

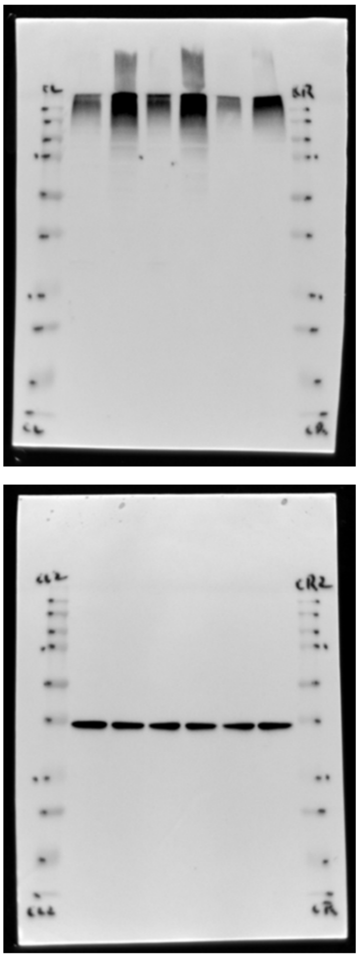

Supplementary Figure 3a

TREM2

TREM2

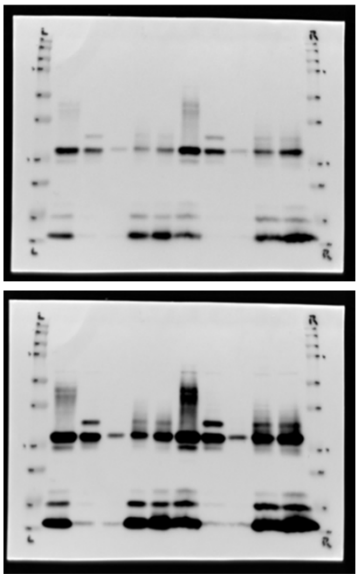

Supplementary Figure 3b

TREM2

TREM2

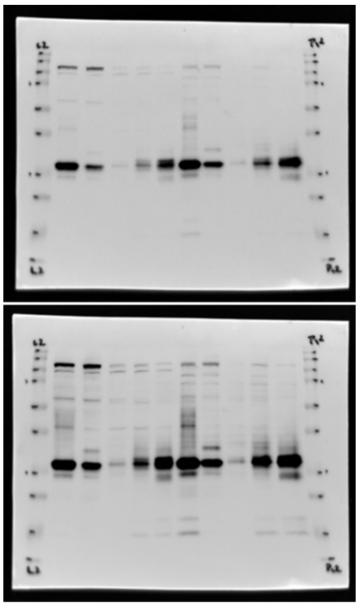

Supplementary Figure 10. Original uncropped images for figures.

Supplementary Figure 10 (continued)

Supplementary Figure 6a

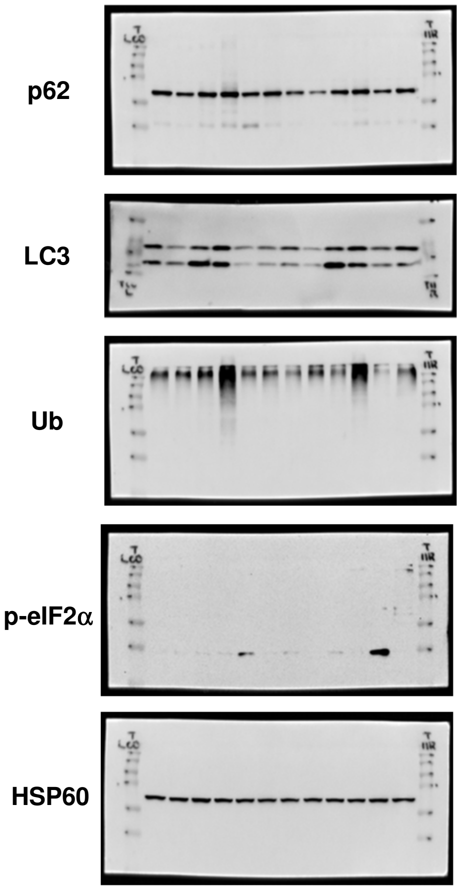

Supplementary Figure 6b

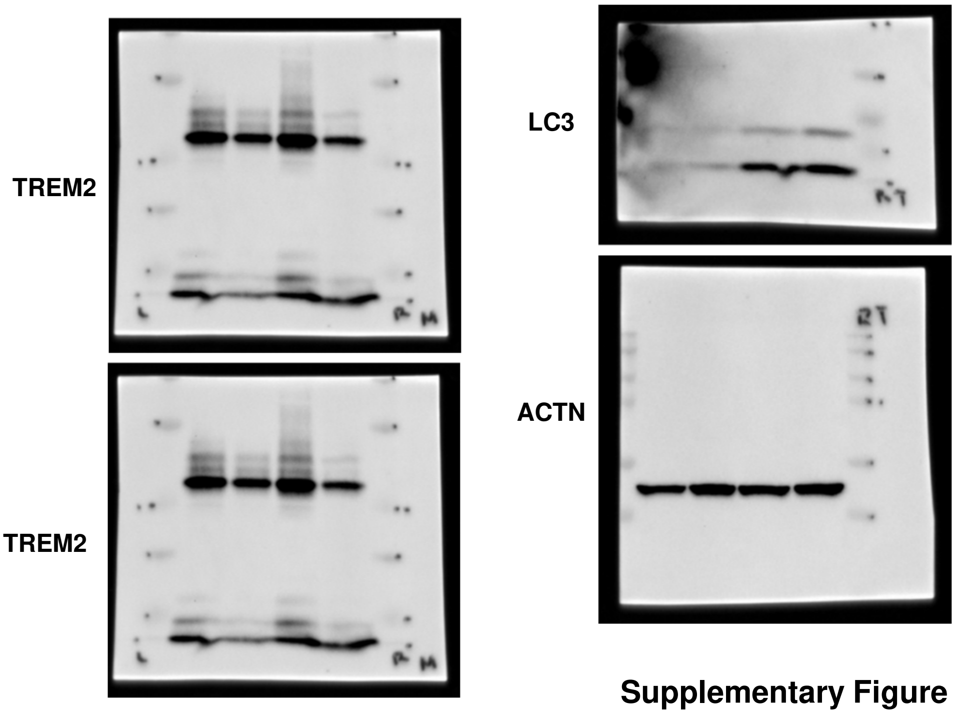

Supplementary Figure 9

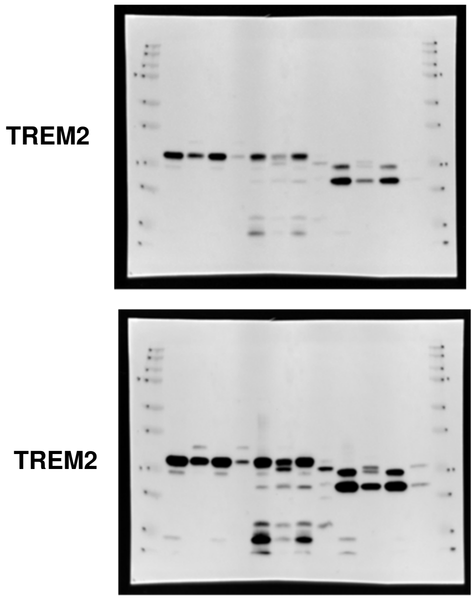

Supplementary Figure 4

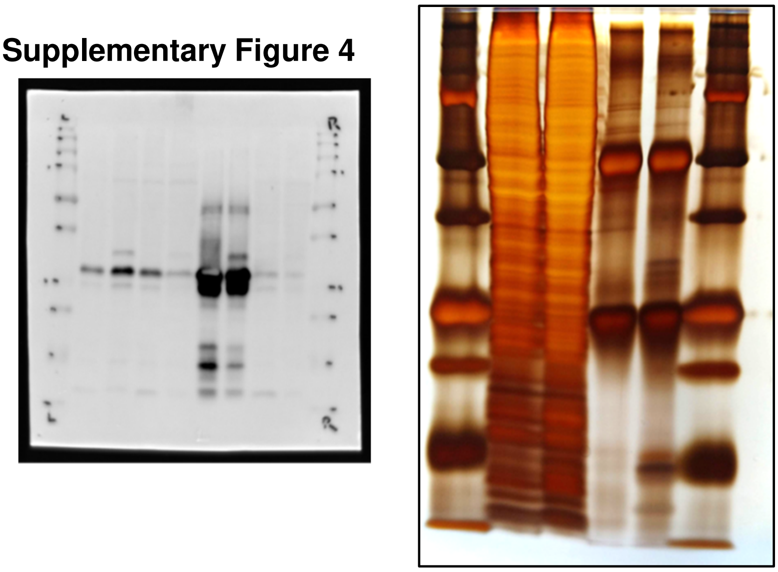

Supplementary Figure 10. Original uncropped images for figures.
