# Supplementary material for: Translational regulation and protein-coding capacity of the 5′ untranslated region of human TREM2
Source: Commun Biol. 2023 Jun 8;6:616. doi: 10.1038/s42003-023-04998-6 (PMC10250343; doi:10.1038/s42003-023-04998-6)
Supplement: Supplementary file 2 — Description of Additional Supplementary Files [file 42003_2023_4998_MOESM2_ESM.pdf]

Description of Additional Supplementary Files

File name: Supplementary Data 1
Description: The source data behind the graphs in this paper.
