# Supplementary material for: Translational regulation and protein-coding capacity of the 5′ untranslated region of human TREM2
Source: Commun Biol. 2023 Jun 8;6:616. doi: 10.1038/s42003-023-04998-6 (PMC10250343; doi:10.1038/s42003-023-04998-6)
Supplement: Supplementary file 4 — Reporting Summary [file 42003_2023_4998_MOESM4_ESM.pdf]

nature portfolio

Corresponding author(s): Yoshihiro Kino
Last updated by author(s): May 17, 2023

# Reporting Summary

Nature Portfolio wishes to improve the reproducibility of the work that we publish. This form provides structure for consistency and transparency in reporting. For further information on Nature Portfolio policies, see our Editorial Policies and the Editorial Policy Checklist.

## Statistics

For all statistical analyses, confirm that the following items are present in the figure legend, table legend, main text, or Methods section.

| n/a | Confirmed | |
|---|---|---|
| ☐ | ☒ | The exact sample size (*n*) for each experimental group/condition, given as a discrete number and unit of measurement |
| ☐ | ☒ | A statement on whether measurements were taken from distinct samples or whether the same sample was measured repeatedly |
| ☐ | ☒ | The statistical test(s) used AND whether they are one- or two-sided<br>*Only common tests should be described solely by name; describe more complex techniques in the Methods section.* |
| ☒ | ☐ | A description of all covariates tested |
| ☒ | ☐ | A description of any assumptions or corrections, such as tests of normality and adjustment for multiple comparisons |
| ☐ | ☒ | A full description of the statistical parameters including central tendency (e.g. means) or other basic estimates (e.g. regression coefficient) AND variation (e.g. standard deviation) or associated estimates of uncertainty (e.g. confidence intervals) |
| ☒ | ☐ | For null hypothesis testing, the test statistic (e.g. *F*, *t*, *r*) with confidence intervals, effect sizes, degrees of freedom and *P* value noted<br>*Give P values as exact values whenever suitable.* |
| ☒ | ☐ | For Bayesian analysis, information on the choice of priors and Markov chain Monte Carlo settings |
| ☒ | ☐ | For hierarchical and complex designs, identification of the appropriate level for tests and full reporting of outcomes |
| ☒ | ☐ | Estimates of effect sizes (e.g. Cohen's *d*, Pearson's *r*), indicating how they were calculated |

*Our web collection on statistics for biologists contains articles on many of the points above.*

## Software and code

Policy information about availability of computer code

| | |
|---|---|
| Data collection | Luminograph III (ATTO)<br>Quadrupole-Orbitrap tandem mass spectrometry (MS) system, Orbitrap Q Exactive MS connected to EASY-nLC 1000 (Thermo Fisher Scientific)<br>LSM710 (Carl Zeiss) |
| Data analysis | Fiji image J (Java 1.8.0_172, 64-bit), EXCEL Toukei software (ESUMI Co., Ltd.), R (version 3.6.1, https://www.r-project.org/) |

For manuscripts utilizing custom algorithms or software that are central to the research but not yet described in published literature, software must be made available to editors and reviewers. We strongly encourage code deposition in a community repository (e.g. GitHub). See the Nature Portfolio guidelines for submitting code & software for further information.

## Data

Policy information about availability of data

All manuscripts must include a data availability statement. This statement should provide the following information, where applicable:

- Accession codes, unique identifiers, or web links for publicly available datasets
- A description of any restrictions on data availability
- For clinical datasets or third party data, please ensure that the statement adheres to our policy

Source data are available in Supplementary Data 1. All other data are available from the corresponding data upon reasonable request.

## Human research participants

Policy information about studies involving human research participants and Sex and Gender in Research.

| | |
|---|---|
| Reporting on sex and gender | No human participantes in this study. |
| Population characteristics | No human participantes in this study. |
| Recruitment | No human participantes in this study. |
| Ethics oversight | No human participantes in this study. |

Note that full information on the approval of the study protocol must also be provided in the manuscript.

# Field-specific reporting

Please select the one below that is the best fit for your research. If you are not sure, read the appropriate sections before making your selection.

☒ Life sciences ☐ Behavioural & social sciences ☐ Ecological, evolutionary & environmental sciences

For a reference copy of the document with all sections, see nature.com/documents/nr-reporting-summary-flat.pdf

# Life sciences study design

All studies must disclose on these points even when the disclosure is negative.

| | |
|---|---|
| Sample size | No statistical methods were used to predetermine sample size. |
| Data exclusions | No data were excluded from the analysis. |
| Replication | Biochemical assays were repeated three times, unless specified otherwise. Consistent resutls were obtained in all instances. |
| Randomization | No studies were conducted that required randomization. |
| Blinding | Data were not treated blindly beucase it is not a common precedure for biochamical assays coducted in this study. |

# Reporting for specific materials, systems and methods

We require information from authors about some types of materials, experimental systems and methods used in many studies. Here, indicate whether each material, system or method listed is relevant to your study. If you are not sure if a list item applies to your research, read the appropriate section before selecting a response.

## Materials & experimental systems

| n/a | Involved in the study | |
|---|---|---|
| ☐ | ☒ | Antibodies |
| ☐ | ☒ | Eukaryotic cell lines |
| ☒ | ☐ | Palaeontology and archaeology |
| ☒ | ☐ | Animals and other organisms |
| ☒ | ☐ | Clinical data |
| ☒ | ☐ | Dual use research of concern |

## Methods

| n/a | Involved in the study | |
|---|---|---|
| ☒ | ☐ | ChIP-seq |
| ☒ | ☐ | Flow cytometry |
| ☒ | ☐ | MRI-based neuroimaging |

## Antibodies

| | |
|---|---|
| Antibodies used | The following antibodies were used for western blot: Goat anti-TREM2 antibody (R&D, pAb, AF1828), Rabbit anti-TREM2 antibody (Cell Signaling Technology, mAb, clone D8I4C, #91068), Rabbit anti-p62/SQSTM1 antibody (proteintech, pAb, #18420-1-AP), Goat anti-HSP60 antibody (Everest biotech, pAb, EB12834), Rabbit anti-Amyloid Precursor Protein antibody (abcam, mAb, clone Y188, ab32136), Mouse anti-GAPDH antibody (MBL, mAb, clone 3H12, #M171-3), Rabbit anti-LC3 antibody (MBL, pAb, PM036), Rabbit anti-phospho-eIF2α antibody (Sigma, pSer51, pAb, SAB4504388), Rabbit anti-Ubiquitin antibody (Dako, pAb, Z0458), Donkey anti-goat IgG (abcam, ab97120), Goat anti-mouse IgG (abcam, ab97040), Goat anti-rabbit IgG (Jackson ImmunoResearch, 111-035-144), Mouse anti-rabbit IgG (ROCKLAND, eB182), normal Goat IgG (santa cruz, sc-2028), Alexa Fluor 488 goat anti-rabbit IgG (H+L) (invitrogen, A11034), Alexa Fluor 568 donkey anti-rabbit IgG (H+L) (invitrogen, A10042). |

| | |
|---|---|
| Validation | The validation is reported in the manufacture's website. |

## Eukaryotic cell lines

Policy information about cell lines and Sex and Gender in Research

| | |
|---|---|
| Cell line source(s) | HEK293 cells: RCB1637, Riken BRC<br>HeLa cells: RCB0007, Riken BRC<br>THP-1 cells: RCB1189, Riken BRC<br>HSCj-110 cells: JCRB1655, JCRB Cell Bank<br>HSP-239 cells: JCRB1165, JCRB Cell Bank<br>RAW264.7 cells: #91062702, ECACC<br>Flp-In-293 cells: Thermo Fisher Scientific<br>Flp-In-293-based fl-TREM2 cells: Yanaizu et al., 2018 |
| Authentication | None of the cell lines were authenticated. |
| Mycoplasma contamination | Cell lines were regularly tested for mycoplasma contamination. |
| Commonly misidentified lines<br>(See ICLAC register) | No commonly misidentified lines were used. |
